# Exosomes and Extracellular Vesicles in Oncology: Next-Generation Smart Nanocarriers for Precision Cancer Therapy

**DOI:** 10.3390/cells15191812

**Published:** 2026-10-04

**Authors:** Mohd Masih Uzzaman Khan, Masood Alam Khan, Mohd Azam, Muhammad Afzal, Tariq G. Alsahli

**Affiliations:** 1 Department of Pharmaceutical Chemistry, College of Pharmacy, Qassim University, Buraydah 51452, Saudi Arabia; 2 Department of Basic Health Sciences, College of Applied Medical Sciences, Qassim University, Buraydah 51452, Saudi Arabia; 3 Department of Medical Laboratories, College of Applied Medical Sciences, Qassim University, Buraydah 51452, Saudi Arabia; m.aftab@qu.edu.sa; 4 Department of Pharmaceutical Sciences, Pharmacy Program, Batterjee Medical College, Jeddah 21442, Saudi Arabia; mohmmad.afzal@bmc.edu.sa; 5 Department of Pharmacology, College of Pharmacy, Jouf University, Sakaka 72341, Saudi Arabia

**Keywords:** exosomes, extracellular vesicles, precision cancer therapy, targeted drug delivery, gene therapy, immunotherapy, tumor microenvironment

## Abstract

Exosomes are an evolved sub-type of extracellular vesicles (EVs) that have gained popularity in recent years as a novel biological nanocarrier system for targeted precision therapy in cancer owing to their excellent biocompatibility, low immunogenicity, and high stability. While other artificial systems can only deliver specific types of drug, exosomes have been found to effectively carry different types of therapeutic cargo, such as chemotherapeutic agents, proteins, peptides, mRNA, siRNA, miRNA, and even CRISPR/Cas gene editing elements. Recent progress in exosome engineering technologies, such as surface modification, donor cell modification, bionic hybrid vesicles, and stimuli-responsive exosomes, has improved their targeting efficacy, cargo-loading capacity, and controlled drug release properties. Apart from their therapeutic use, exosomes play an active role in promoting tumor development by regulating the tumor microenvironment, angiogenesis, metastasis, immune escape, maintenance of cancer stem cells, and drug resistance. This review provides a brief insight into the biological properties of exosomes, recent developments in isolation and characterization methods, engineering approaches, therapeutic cargo loading, and their uses in targeted chemotherapy, gene and RNA therapy, immunotherapy, phototherapy, sonodynamic therapy, chemo-dynamic therapy, and combinational therapy. Moreover, the existing issues in exosome research, such as exosome heterogeneity, large-scale production, quality control, and regulatory issues, are addressed. Finally, new approaches involving artificial intelligence, multi-omics technology, and personalized engineering of exosomes are presented as potential solutions to enhance the clinical use of exosomes.

## 1. Introduction

Cancer continues to be one of the major causes of death and disease burden, despite tremendous progress made in its early detection, molecular profiling, and management through treatment protocols [1]. Conventional cancer treatment therapies, such as surgery, chemotherapy, radiation therapy, targeted therapy, and immunotherapy, have greatly enhanced the survival rate of patients. Nevertheless, these treatments fail due to lack of tumor specificity, toxicity, drug resistance, and lack of penetration into the tumor microenvironment (TME) [2]. Tumor heterogeneity, the interaction between cancer cells and the stroma, and the suppressive environment of TME, are some of the additional problems that prevent successful treatment. There is a dire need to develop innovative drug delivery systems that will selectively target tumors while avoiding off-targets [3]. The emergence of nanotechnology in cancer treatment has resulted in the advancement of nanocarriers, which provide enhanced pharmacokinetics, increased stability of the drug, and controlled release of the drug [4]. Synthetic nanocarriers, including liposomes, lipid nanoparticles, polymeric nanoparticles, and dendrimers, have demonstrated substantial utility in drug delivery because their composition, physicochemical properties, surface characteristics, and drug-release profiles can be systematically controlled [5]. Their relatively well-defined manufacturing processes and scalable production also facilitate formulation optimization and quality control. However, depending on their composition and surface characteristics, synthetic nanocarriers may face challenges related to immune recognition, rapid clearance, limited tumor accumulation, or biological barrier penetration [6]. In contrast, biological extracellular vesicles (EVs) possess naturally derived lipid membranes and complex molecular compositions that may support cellular interactions, cargo protection, and biological communication [7]. Nevertheless, EV-based systems have their own limitations, including source-dependent heterogeneity, complex cargo composition, batch-to-batch variability, challenges in isolation and purification, and difficulties in achieving standardized large-scale manufacturing [8]. Therefore, EVs and synthetic nanocarriers should be considered complementary delivery platforms, with their suitability depending on the therapeutic cargo, target tissue, manufacturing requirements, and intended clinical application [9]. The above limitations have prompted more research into natural nanocarriers with biologically functional properties [10]. For these natural systems, among others, the next-generation smart nanocarriers have already arrived in terms of precision oncology; exosomes are natural biological EVs [11]. These lipid bilayer–enclosed vesicles are released from nearly all kinds of cells through the endosomal pathway and deliver a complex set of bioactive molecules, such as proteins, lipids, DNA, messenger RNA (mRNA), microRNA (miRNA), long non-coding RNA (lncRNA), circular RNA (circRNA), and other metabolites, to recipient cells, modulating cell-cell communications [12]. Exosomes possess distinctive biological properties that support their investigation as naturally derived delivery systems, although their performance remains dependent on source, composition, and preparation conditions [13]. Initially thought of as waste-disposal vesicles for the cell, exosomes have since been discovered to be very significant in the mediation of physiological and pathological phenomena. In a normal state, exosomes regulate the immune response, angiogenesis, tissue repair, stem-cell maintenance, and cellular homeostasis. Nevertheless, in cancer, cancer-derived exosomes are involved in the progression of the disease through mediating interactions between cancer cells and their microenvironment [14]. As the result of oncogenic protein and nucleic acid exchange, they contribute to tumorigenesis, epithelial-mesenchymal transition, angiogenesis, remodelling of the extracellular matrix, immune escape, pre-metastatic niche formation, and resistance to both chemotherapy and immunotherapy. At the same time, engineered and immune or stem cell-derived exosomes show high therapeutic efficiency due to delivery of anticancer drugs, nucleic acids, proteins, and gene-editing systems to cancer tissues [15]. The recent developments in the field of bioengineering have greatly increased the range of applications for exosomes. Surface modifications, genetic manipulation of donor cells, membrane fusion, and chemical conjugations have made it possible to obtain smart exosomes, which show high targeting capacity and cargo delivery control [16]. In addition, new methods of loading such as electroporation, sonication, extrusion, microfluidic technique, and cargo engineering within exosomes have been developed for improving the loading of chemotherapeutics, siRNAs, miRNAs, mRNAs, CRISPR/Cas systems, proteins, and peptides into exosomes. Combination of exosomes and artificial intelligence, omics, and precision medicine has additionally facilitated the development of personalized therapies [17]. In addition to being used in the treatment, exosomes have become useful biomarkers for both diagnostics and prognosis due to their stability in various biological fluids such as blood, urine, saliva, and cerebrospinal fluid. The molecules carried by exosomes perfectly mirror the physiological status of parent cells, thus making them very good candidates for use in liquid biopsies, early diagnosis of cancer, treatment monitoring, and treatment response prediction [18]. However, obstacles such as large-scale production, isolation techniques standardization, heterogeneous cargos, quality control, storage stability, and regulatory issues still prevent clinical translation [19]. This review provides an extensive overview on the biology of exosomes and EVs, their diverse role in the process of cancer development, latest developments in the field of engineering, drug delivery into them, precision oncology, TME targeting, theranostics, and artificial intelligence-assisted technologies. In addition to that, the challenges of translation and future prospects are analyzed to demonstrate the enormous potential of exosomes as next-generation smart nanocarriers for cancer therapy [20].

## 2. Biological Fundamentals of Exosomes and EVs

**Figure 1:**
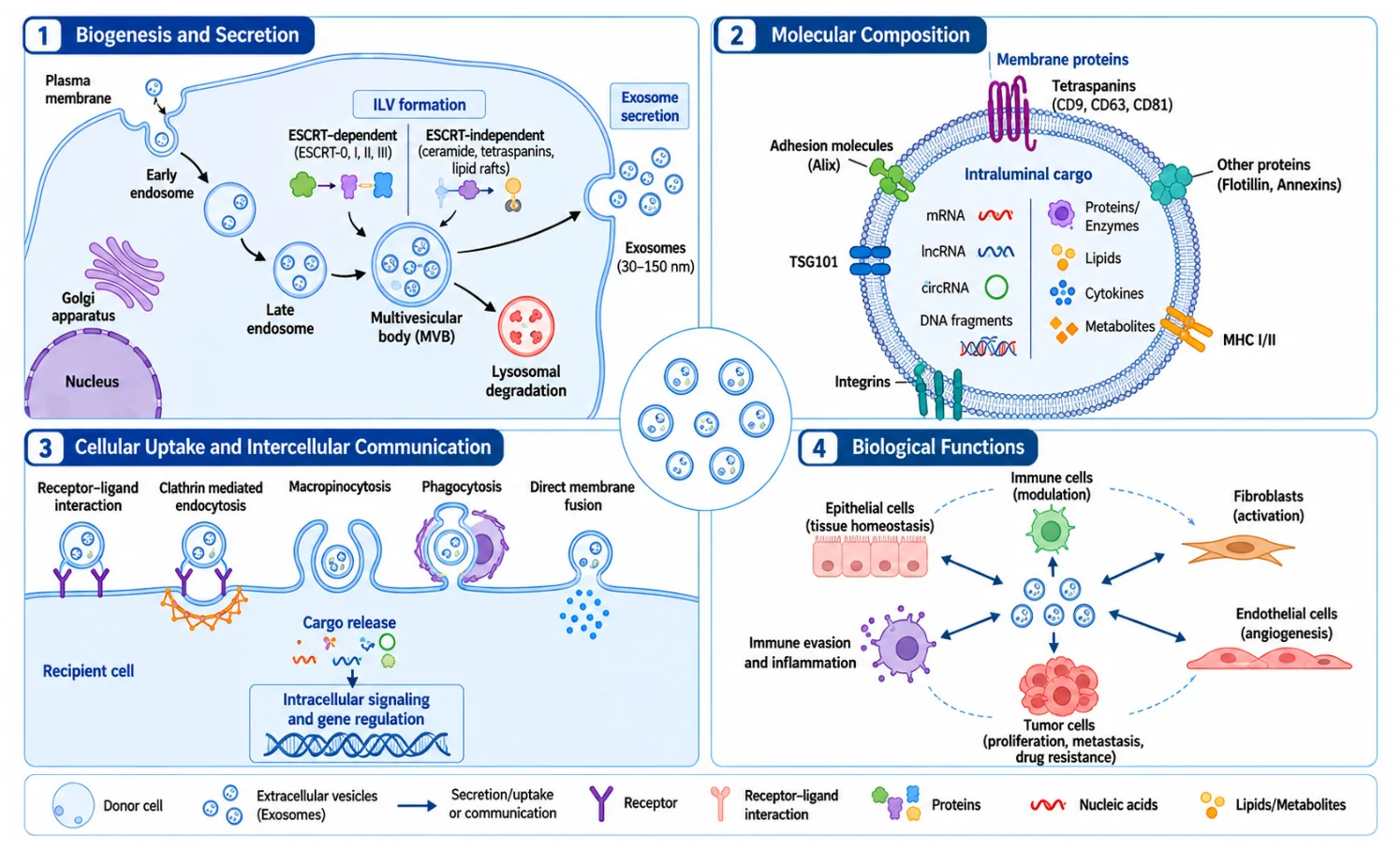
Exosome biogenesis, composition, uptake, and biological functions. Schematic representation of exosome formation and secretion, molecular cargo, mechanisms of cellular uptake and intercellular communication, and their major roles in regulating immune, epithelial, fibroblast, endothelial, and tumor cell functions.

EVs are nanoscale structures that have a phospholipid bilayer surrounding them and are secreted by practically all types of cells into their surrounding environment. Initially, EVs were regarded as waste products created by cells, however, today, it is known that they play a crucial role in intercellular signalling and are involved in various biological processes [21]. These particles contain a wide range of bioactive molecules, such as proteins, lipids, DNA, mRNA, miRNA, lncRNA, circRNA, metabolites, and signalling molecules, and therefore influence the activities of recipient cells. The content of EVs represents the physiological/pathological status of their source cells [22]. In cancer, EVs originating from tumors play a significant role in tumor progression, angiogenesis, immune escape, metastasis, and drug resistance by transporting oncoproteins to nearby and remote cells. In contrast, artificially produced EVs and exosomes secreted by stem cells have been investigated as biologically derived nanocarriers for drug delivery because of their favorable biocompatibility, potential for prolonged circulation, and ability to interact with recipient cells through naturally occurring surface molecules [23]. However, natural cellular tropism does not necessarily indicate tumor-specific targeting, which generally requires experimental validation or additional surface engineering. Biological aspects of exosomes include exosome formation, composition, communication between cells, and biological activities that define their physiological and pathophysiological significance. Biological processes that constitute the biology of exosomes are presented in Figure 1.

### 2.1. Classification of EVs

EVs comprise a heterogeneous population of lipid bilayer-enclosed particles released by cells and differ in their biogenesis, size distribution, molecular composition, cellular origin, and biological functions [24]. Although EVs have traditionally been classified into exosomes, microvesicles (ectosomes), and apoptotic bodies according to their presumed biogenesis and physical characteristics, such classification cannot always be established reliably in experimentally isolated preparations because substantial overlap exists in their size, molecular composition, and surface markers [25]. Therefore, current EV nomenclature emphasizes the need to describe vesicles according to experimentally supported characteristics rather than assigning a specific biogenesis-based subtype solely from size or commonly detected markers [26].

The term exosome is generally used for EVs of endosomal origin that are released through the multivesicular endosomal pathway. However, commonly used markers such as CD9, CD63, CD81, ALIX, and TSG101 are associated with many EV preparations and are not, individually or collectively, sufficient to establish an endosomal origin [27]. Similarly, a size range of approximately 30–150 nm should not be considered a definitive criterion for identifying exosomes because EV populations show substantial overlap in size [28]. Microvesicles, also referred to as ectosomes, are generally associated with outward budding and fission of the plasma membrane, whereas, apoptotic bodies are larger vesicular structures generated during apoptotic cell disassembly [29]. The term EV is used as a broader designation, whereas the term exosome is used specifically when an endosomal origin is demonstrated or when discussing studies that explicitly characterize their vesicles as exosomes [30,31]. Exosomes and other small EVs have been extensively investigated as potential therapeutic carriers because of their biocompatibility, complex surface composition, and ability to interact with recipient cells, however, their natural cellular tropism should not be equated with universal or tumor-specific targeting [32]. Microvesicles are formed through the direct outward budding of the plasma membrane by means of cytoskeletal rearrangement and calcium signalling. They carry proteins such as receptors, enzymes, and signalling molecules that participate in inflammation, angiogenesis, immune regulation, and tumor development. Apoptotic bodies are formed through the process of programmed cell death. They consist of cell organelles, DNA, and cytoplasmic contents to help with the removal of dead cells and maintain tissue homeostasis [33]. EVs are a diverse set of membrane-enclosed particles with distinct biogenesis pathways, sizes, molecular compositions, and biological functions. We present a comparative synopsis of the primary classes of EVs: exosomes, microvesicles, apoptotic bodies, large oncosomes, and exomeres (Table 1). It is also important to distinguish EVs from other extracellular particles that may co-isolate with EV preparations. Exomeres and supermeres have been described as non-vesicular extracellular particles and should therefore not be classified as conventional EV subtypes. These particles may co-exist with or co-isolate alongside EVs during isolation procedures, highlighting the importance of appropriate separation and characterization when studying complex extracellular particle preparations.

### 2.2. Biogenesis and Secretion Pathways

Biogenesis pathways of different subtypes of EVs vary. In cells, the biogenesis pathway of exosomes is initiated through invagination of the plasma membrane, forming an early endosome, which matures into a late endosome [38]. The endosomal membrane can inward bud to produce ILVs, which assemble into MVBs. ILVs are then selectively sorted into MVBs, and these cargoes include lipids, proteins, and nucleic acid molecules mediated by ESCRT-dependent (Endosomal Sorting Complex Required for Transport) and ESCRT-independent pathways, such as ceramide, tetraspanin, and lipid rafts. Finally, MVBs fuse either with lysosomes to be degraded or with the plasma membrane, secreting ILVs to the extracellular space as exosomes regulated by Rab GTPases (Rab27a, Rab27b, Rab11, and Rab35) and SNARE proteins [39]. In contrast, microvesicles develop via outward budding of the plasma membrane, due to remodelling of the cytoskeleton, redistribution of phospholipids, and Ca^2+^-mediated signalling. Apoptotic bodies are formed during programmed cell death due to blebbing and fragmentation of cells [40]. Hypoxia, oxidative stress, inflammation, and oncogenic signals are some of the environmental signals that affect EV formation and loading with cargo. In cancers, deregulation of such pathways causes increased secretion of EVs, resulting in tumor growth, metastases, immune escape, drug resistance, and exosome engineering as therapeutic carriers [41].

### 2.3. Molecular Composition and Structural Characteristics

Molecular characteristics serve as biosignatures of the physiological state and cellular source of the parent cell EVs, which are lipid bilayers constituted of cholesterol, sphingomyelin, ceramide, and phosphatidylserine. These components confer stability to EVs in body fluids and protect their contents against enzymatic degradation [31]. EVs comprise multiple categories of bioactive biomolecules, including proteins, lipids, DNA, messenger RNA (mRNA), microRNA (miRNA), long non-coding RNA (lncRNA), circular RNA (circRNA), metabolomics, and enzymes [42]. EV membranes commonly contain proteins such as CD9, CD63, CD81, ALIX, TSG101, flotillins, heat shock proteins (HSP70/HSP90), and integrins. Although several of these proteins are frequently detected in exosome-enriched preparations, their presence alone does not establish an endosomal origin or provide definitive evidence that a preparation consists exclusively of exosomes [43]. Cancer exosome cargos may consist of oncogenes, growth factors, immune checkpoints, and modulating RNAs that facilitate cancer initiation, development, angiogenesis, metastasis, and drug resistance. The structural stability and special molecular content in EVs can thus provide opportunities for application of the vesicles as tumor biomarkers in liquid biopsy and natural carriers of drugs for precision oncology [23].

### 2.4. Mechanisms of Cellular Uptake and Intercellular Communication

Intracellular communication via EVs occurs through the transportation of cargos from one cell to another through different uptake mechanisms of EVs. Following their release, EVs interact with target cells through processes, including ligand-receptor binding, membrane fusion, and different types of endocytosis, such as clathrin-dependent endocytosis, caveolae-dependent endocytosis, micropinocytosis, and phagocytosis [44,45]. After uptake into cells, the contents of EVs, such as proteins, lipids, DNA, mRNA, miRNAs, and other types of non-coding RNAs, are delivered to the cytosol, which regulates gene expression and intracellular signalling and metabolic pathways. Cargo delivery affects many physiological events, such as proliferation, differentiation, angiogenesis, immune function, and tissue healing [46]. In cancer, tumor-originated EVs facilitate intensive communication among the TME through interactions with cancer cells, fibroblasts, endothelial cells, and immune cells. This results in cancer progression, angiogenesis, epithelial to mesenchymal transition, immune escape, formation of metastatic niches, and drug resistance. Conversely, engineered exosomes use the natural communication routes for selective drug delivery, thus, EVs hold great potential as nanocarriers in cancer treatment [47].

### 2.5. Role of EVs in Physiological and Pathological Processes

EVs play an extremely important role in the physiological maintenance of biological homeostasis due to their contribution towards cell-to-cell communication and the exchange of biologically active compounds. In healthy individuals, EVs regulate immune system functions, tissue repair, formation of new blood vessels, stem cell functions, neural communication, inflammation, and cellular differentiation [48]. Pathological states, such as cancer, profoundly affect the secretion and composition of EVs. EVs produced by tumor cells accelerate cancer development by delivering oncogenic proteins, RNA, and DNA, as well as signalling molecules, which cause increased tumor proliferation, angiogenesis, EMT, immune escape, extracellular matrix remodelling, metastasis, chemoresistance, and immuno-resistance. Furthermore, EVs affect the tumor microenvironment (TME) by affecting immune cells, fibroblasts, and endothelial cells to facilitate tumor growth and spread [49]. Apart from their involvement in the development of diseases, EVs have shown potential as biomarkers and a means to deliver drugs because they carry cargo that is characteristic of the originating cells. Beyond their biological roles, EVs are being investigated as biomarkers and delivery platforms because their molecular cargo can reflect the characteristics of the originating cells [50].

## 3. Exosomes in Cancer Biology and Tumor Progression

**Figure 2:**
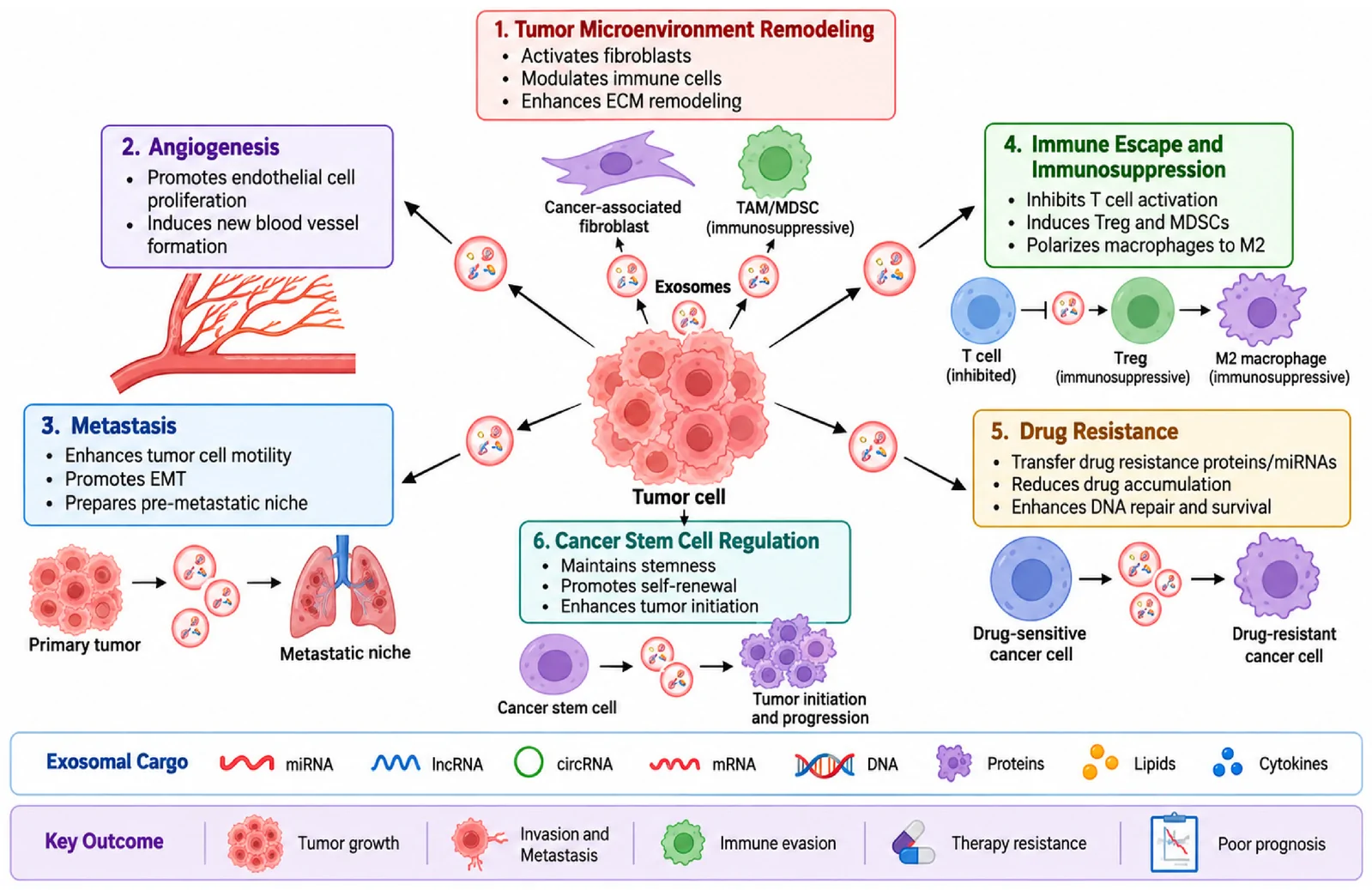
Overview of mechanisms associated with exosomes promoting cancer progression such as remodeling of the tumor microenvironment, angiogenesis, metastasis, immunosuppression, drug resistance and maintenance of cancer stem cells.

Cancer development is now considered a dynamic and interactive process in which tumor progression relies on constant communication between cancer cells and the TME, rather than being solely dependent on genetic changes. As far as the mediators of communication are concerned, tumor derived exosomes (TEXs) are being recognized as important regulators of cancer cell proliferation and progression [51]. Exosomes are carriers of a broad spectrum of cargo comprising proteins, lipids, DNA, mRNA, miRNA, lncRNA, and circRNAs, allowing oncogenic signalling transmission to recipient cells. The composition of their cargo is affected by various factors, including hypoxia, inflammation, oxidative stress, and therapy pressure, which enable tumors to adapt to their surroundings. Through the activation of several signalling pathways, such as PI3K/Akt/mTOR, MAPK/ERK, Wnt/β-catenin, TGF-β/Smad, NF-κB, and JAK/STAT3, exosomes stimulate tumor angiogenesis, EMT, immune escape, extracellular matrix remodelling, metastasis, and therapy resistance [52,53]. Exosomal communication is one of the key processes that drive cancer development by participating in tumor initiation, reprogramming of the tumor microenvironment, angiogenesis, metastasis, immunosuppression, drug resistance, and cancer stem cell survival through molecular transfer. The main mechanisms involved are briefly described in Figure 2.

### 3.1. Sources and Consequences of EV Heterogeneity

EV populations exhibit substantial biological and methodological heterogeneity, which can influence their composition, biological activity, therapeutic performance, and reproducibility. One important source of heterogeneity is the donor cell type and physiological state. EVs released by different cell types can differ in their membrane proteins, lipids, nucleic acids, and protein cargo [54]. Furthermore, changes in cellular activation, proliferation, differentiation, or stress conditions may alter EV production and cargo composition. In cancer, heterogeneity between tumor subtypes can further influence EV characteristics because cancer cells with different genetic and phenotypic profiles may produce vesicles with distinct molecular cargo and biological functions [55].

Cell culture conditions can also affect EV characteristics. Parameters such as culture medium, serum supplementation, cell density, passage number, oxygen availability, and culture duration may influence EV release and molecular composition [56]. In particular, hypoxic conditions and therapeutic treatments can modify cellular signalling and consequently alter the quantity and cargo of released EVs. Such changes may affect the biological activity of EV preparations and should therefore be considered when comparing results between studies [57].

Methodological factors also contribute substantially to EV heterogeneity. Isolation and purification procedures, including differential ultracentrifugation, density-gradient centrifugation, size-exclusion chromatography, precipitation, ultrafiltration, and affinity-based methods, differ in their recovery, purity, and ability to enrich particular EV populations [58]. Consequently, preparations obtained using different methods may contain different proportions of EV subpopulations as well as co-isolated non-EV components. Similarly, variation in EV size distribution and cargo composition can contribute to differences in biological activity and therapeutic performance [59].

Post-isolation processing can introduce additional variability. Storage conditions, freeze–thaw cycles, concentration procedures, buffer composition, and other handling steps may affect EV membrane integrity, aggregation, cargo stability, and biological activity. Therefore, differences in sample preparation and processing can contribute to apparent discrepancies between experimental studies, even when similar cellular sources are used [60].

EV heterogeneity has important implications for therapeutic development. Differences in EV composition and surface characteristics may influence cellular uptake, tissue distribution, pharmacokinetics, targeting efficiency, and therapeutic efficacy. Variations in cargo may also alter biological responses and potentially contribute to differences in toxicity or off-target effects [61]. Moreover, source- and process-dependent variability can make it difficult to reproduce therapeutic outcomes between batches and laboratories. Standardized donor-cell conditions, isolation procedures, characterization criteria, storage conditions, and quality-control parameters are, therefore, important for improving the reproducibility and translational development of EV-based therapies [62]. EV heterogeneity arises from multiple biological and methodological factors that can affect EV characteristics and therapeutic outcomes, as shown in Table 2.

### 3.2. Exosome-Mediated Tumor Communication

Tumor-derived exosomes (TEXs) are highly effective communication mediators that transfer bioactive molecules that help reprogram recipient cells. Once released into the environment, TEXs can be internalized by recipient cells via interaction between receptors and ligands, membrane fusion, or other routes of entry, thus allowing the transport of oncogenic proteins, lipids, and regulatory RNAs. miR-21 and miR-155, which are found in TEXs, downregulate the expression of tumor suppressors such as PTEN and SOCS1, resulting in the activation of PI3K/Akt, NF-κB, and STAT3 signalling pathways [73]. Exosomal TGF-β, EGFR, and MET also exert their effects on CAFs and endothelial cells, causing remodelling of the extracellular matrix, angiogenesis, and formation of metastatic niches. In a hypoxic state, the role of HIF-1α becomes prominent, which not only helps in promoting exosome release but also increases the presence of pro-angiogenic miRNAs within these exosomes. Overall, these processes help in developing a tumor-supportive microenvironment [74].

### 3.3. Tumor Microenvironment Remodelling

In fact, tumor exosomes (TEXs) make a major contribution to the restructuring of the TME by remodelling the phenotypes of stroma, immune, and endothelial cells for tumor progression. Indeed, the content of TGF-β, miR-21, miR-1247-3p, miR-155, VEGF, and MMP-2/MMP-9 promotes activation of fibroblasts by stimulation of the TGF-β/Smad and NF-κBsignalling pathway and their transformation into cancer-associated fibroblasts (CAFs) [75]. These activated fibroblasts secrete extracellular matrix (ECM) components, cytokines (IL-6, CXCL12), and growth factors that stimulate tumor growth and invasion. On the other hand, exosomal VEGF and angiogenic miRNAs promote stimulation of endothelial cells and their activation by means of the PI3K/Akt and ERK1/2 signalling pathways, which contributes to angiogenesis and provision of the tumor with nutrients. At the same time, tumor exosomes lead to the polarization of macrophages in the pro-tumorigenic M2 type via STAT3 signalling [76,77].

### 3.4. Angiogenesis and Metastatic Cascade

Exosomes play an important role in the mediation of angiogenesis and metastasis, allowing tumor spread through molecular signalling. In response to hypoxia, stabilization of HIF-1α promotes exosome release and increases the loading of exosomes with pro-angiogenic factors, such as VEGF, angiopoietin-2, miR-210, miR-23a, and miR-105 [78]. The PI3K/Akt, MAPK/ERK, and VEGFR2 signalling pathways become activated in endothelial cells as a result of the action of the above-mentioned factors, leading to cell proliferation, migration, and formation of new blood vessels [79]. At the same time, exosomal MMP-2, MMP-9, and integrins lead to degradation of the ECM and EMT in the result of activation of the TGF-β/Smad and Wnt/β-catenin signalling pathways. Moreover, organ-specific integrins of the exosomes of tumor origin mediate the process of the pre-metastatic niche formation by recruiting bone marrow cells [80].

### 3.5. Immune Escape and Immunosuppression

Tumor exosomes are crucial mediators in creating the immunosuppressive microenvironment of tumors by interfering with both the innate and adaptive immune systems. TEXs contain immunomodulatory molecules such as PD-L1, TGF-β, IL-10, Fas ligand (FasL), and immunosuppressive microRNA (miR-21 and miR-146a) that inhibit the activation and cytotoxicity of CD8^+^ T cells and natural killer (NK) cells [81]. Exosomal PD-L1 interacts with the PD-1 receptor on the surface of T cells, leading to the inhibition of T cell receptor (TCR) signalling and T cell exhaustion [82]. Meanwhile, exosomal TGF-β and IL-10 lead to the expansion of regulatory T cells (Tregs) and polarization of macrophages into the M2 phenotype due to the activation of STAT3 and Smad signalling pathways [83].

### 3.6. Drug Resistance Mechanisms

The importance of exosomes in developing resistance to treatment is becoming more apparent in the sense that exosomes promote horizontal gene transfer of drug-resistant phenotypes. Exosomes contain drug efflux transporters P-glycoproteins and ABC transporters, anti-apoptotic proteins, oncogene mutations, and regulatory RNA molecules [84]. Exosomal miR-21, miR-222, miR-100, and miR-155 downregulate tumor suppressor proteins PTEN and PDCD4, resulting in the activation of survival pathways PI3K/Akt/mTOR, MAPK/ERK, and NF-κB [85]. the cell-secreted exosomes also serve as chemotherapy agents’ vehicle, and can result in reduction in the concentration of the drugs in the cytoplasm while exosomal PD-L1 promote immune checkpoint inhibitors resistance through inhibiting T cell response. Hypoxia-induced exosomes enhance resistance through EMT, metabolic remodelling, and plasticity of cancer cells [86].

### 3.7. Cancer Stem Cell Regulation

Cancer stem cells (CSCs) are tumorigenic cells that serve as the origin of tumors, contributing to metastasis, recurrence, and therapeutic resistance. Tumor-derived exosomes (TEXs) maintain CSC self-renewal and plasticity by transferring various onco-proteins and regulatory RNA molecules, including miR-21,miR-155, and miR-221, which induce Wnt/β-catenin, Notch, Hedgehog, PI3K/Akt, and STAT3 pathway activation, leading to improved self-renewal, epithelial-to-mesenchymal transition (EMT), self-renewal, resistance to apoptosis, maintenance, and growth [87]. The exosomes derived from CSCs modulate the microenvironment of the tumor through stimulation of fibroblasts, endothelial cells, and immune cells, leading to the release of IL-6, CXCL12, VEGF, and TGF-β in order to develop a microenvironment that favors the existence and proliferation of CSCs. In the hypoxic state, exosome secretion is increased, which leads to tumor heterogeneity and resistance to various treatments, such as chemotherapy, radiotherapy, and immunotherapy [88].

## 4. Isolation, Purification, and Characterization Strategies

The clinical translation of EVs as therapeutic agents hinges on the development of reliable, reproducible, and scalable methods for their isolation and characterization. Due to the presence of proteins, lipoproteins, nucleic acids, and other extracellular particles in addition to EVs in biological fluids, the isolation of pure vesicle preparations continues to be one of the most significant challenges [89]. Ideally, a good method will be able to ensure the preservation of vesicle integrity and functionality, high yields, high purity, reproducibility, and compatibility with further downstream analysis. At present, there is no method that satisfies all of these criteria simultaneously, therefore, the choice of the right method is largely sample-dependent [90].

### 4.1. Conventional Isolation Techniques

Traditional isolation techniques continue to form the core of EV studies owing to their well-defined techniques and availability. The DUC technique is considered the gold standard and works by separating vesicles according to their size and density via centrifugation. Although DUC produces relatively pure exosomes, the procedure is tedious, expensive, and time-consuming [33]. Other commonly utilized techniques include density-gradient ultracentrifugation, size exclusion chromatography (SEC), precipitation using polymers, and ultrafiltration. Density gradient centrifugation is the most efficient technique for obtaining purity because it effectively separates EVs from proteins, although it has a very limited throughput capacity. SEC ensures vesicle structure integrity and biological functionality while maintaining protein purity; hence, it is more favored for clinical purposes [91]. On the other hand, precipitation techniques ensure high recovery and ease of use but end up isolating plasma proteins and lipoproteins in addition to EVs, lowering the purity of the sample. Ultrafiltration helps in quickly concentrating EVs, although there is a risk of mechanical damage to the membranes [92].

### 4.2. Advanced Isolation Technologies

New technologies developed in the field of nanotechnology and bioengineering include highly selective isolation strategies that can address the shortcomings of traditional technologies. The use of these technologies is based on differences in surface biomarkers, electrochemical characteristics, acoustic features, and microfluidic dynamics. Advanced technologies require smaller sample amounts, less processing time, and better reproducibility than traditional technologies [93]. New techniques provide the ability to isolate EVs according to disease-specific biomarkers that exist within those EV subpopulations. Although most such technologies are still under development, they represent an important milestone for automation and standardization of EV isolation procedures [94].

### 4.3. Microfluidics

The development of microfluidics has changed the process of EV isolation, where sample preparation, isolation, and analysis are performed in miniaturized labs-on-a-chip. The principle of microfluidic technology involves the manipulation of fluids in tiny channels, which enables fast isolation from small sample volumes without compromising the structure of vesicles. The principle of isolation can be based on various parameters [95]. In contrast to ultracentrifugation, microfluidics provides advantages such as fast processing, reduced reagent usage, high reproducibility, and ease of automation. In addition, their compatibility with biosensors and molecular detection systems allows simultaneous isolation and biomarker identification, making them promising for liquid biopsies. However, the difficulty in fabrication, reduced processing volume, and mass production are some of the limitations that currently exist [96].

### 4.4. Immunoaffinity Capture

Immunoselection is based on the use of antibodies raised against the surface proteins of exosomes, such as CD9, CD63, CD81, EpCAM, HER2, and EGFR. Antibodies coupled to magnetic beads, microplates, and functional nanoparticles allow for highly selective enrichment of disease-specific exosomes [97]. This method is highly pure and is very useful in identifying cancer biomarkers because only cancer-derived exosomes can be selectively isolated. Unfortunately, there is always lower yield with immunologic isolation, it is costly, and also not all EV sub-populations will be captured because not all have the antibodies. Therefore, this method is commonly coupled with other isolation procedures [98].

### 4.5. Acoustic Separation

Acoustic sorting has recently been described as a label-free isolation technology that utilizes acoustic standing waves to isolate EVs without any chemical or physical reagent. The acoustic force acts on the particle based on its properties, such as size, density, and compressibility, without compromising membrane integrity and functionality. This is a less destructive method compared to ultracentrifugation, as it does not cause aggregation [99]. Several studies have shown that there is huge scope for acoustic microfluidic systems to be used to continuously isolate EVs from blood and other body fluids in an automated fashion. Although the technology has many benefits, it is still at a nascent stage and needs to be optimized [100].

### 4.6. Lab-on-Chip Systems

The lab-on-a-chip (LOC) technology integrates microfluidics, biosensors, immunocapture, and molecular detection into a small device that can perform several experiments simultaneously. The ability of LOC technology to carry out the above-mentioned operations efficiently reduces processing time and laboratory infrastructure [101]. Nanotechnology and AI-powered imaging techniques and electrochemical biosensors have further improved the sensitivity and performance of LOC devices. With such developments, LOC devices have emerged as a powerful technology for real-time liquid biopsy, early cancer detection, therapeutic monitoring, and personalized medicine. Although large clinical validations and regulations are needed for such technologies, LOC devices are anticipated to become an integral part of the clinical translation of exosome-based diagnostics and therapeutics in the future [102]. EV isolation methods vary considerably in terms of yield, purity, reproducibility, sample compatibility, and their ability to selectively enrich specific EV subpopulations. These differences can influence the composition, quality, and downstream therapeutic performance of isolated EV preparations, as shown in Table 3.

## 5. Engineering Strategies for Smart Exosome-Based Nanocarriers

Therapeutic applications of native EVs can be limited by heterogeneous cargo composition, variable targeting efficiency, limited loading capacity for certain therapeutic agents, and difficulties associated with reproducible large-scale manufacturing. Consequently, multiple engineering strategies have been developed to improve EV stability, targetability, cargo loading, bioavailability, and therapeutic performance [112]. Although EVs possess biologically derived membranes and endogenous cellular-interaction properties, their complex composition and source-dependent variability can complicate standardization and manufacturing. In contrast, synthetic nanocarriers, such as liposomes, lipid nanoparticles, and polymeric nanoparticles, generally provide greater control over composition, physicochemical properties, cargo loading, surface modification, and production processes. These characteristics can facilitate reproducibility, scalability, and quality control, although synthetic systems may require additional optimization to achieve efficient cellular uptake, prolonged circulation, and tissue-specific targeting [113].

Therefore, native EVs and engineered EVs represent complementary platforms rather than universally superior alternatives to synthetic nanocarriers. Native EVs may provide biological compatibility and endogenous cell-interaction mechanisms, whereas engineered EVs can be modified to improve targeting and cargo delivery. Meanwhile, synthetic nanocarriers offer greater control over formulation composition and manufacturing parameters [114]. EV-based and synthetic nanocarriers differ substantially in their composition, biological compatibility, targeting properties, cargo-loading capacity, manufacturing control, and translational challenges. Comparing these characteristics helps highlight the complementary advantages and limitations of native EVs, engineered EVs, liposomes, lipid nanoparticles, and polymeric nanocarriers for therapeutic delivery, as shown in Table 4.

Overall, EV-based and synthetic nanocarriers possess distinct strengths and limitations. Native and engineered EVs provide biologically derived membranes and complex molecular features that may facilitate cellular interactions and protection of therapeutic cargo, however, their heterogeneity and source-dependent composition can complicate reproducibility and large-scale manufacturing. Synthetic systems, including liposomes, lipid nanoparticles, and polymeric nanoparticles, generally allow greater control over composition, physicochemical characteristics, surface modification, and production processes. However, their biological performance, including circulation, cellular uptake, tissue distribution, and immune interactions, remains dependent on formulation-specific properties. Thus, the selection of an EV-based or synthetic nanocarrier should be guided by the therapeutic cargo, target tissue, desired pharmacokinetic and release characteristics, manufacturing requirements, and clinical application rather than assuming universal superiority of one platform.

**Figure 3:**
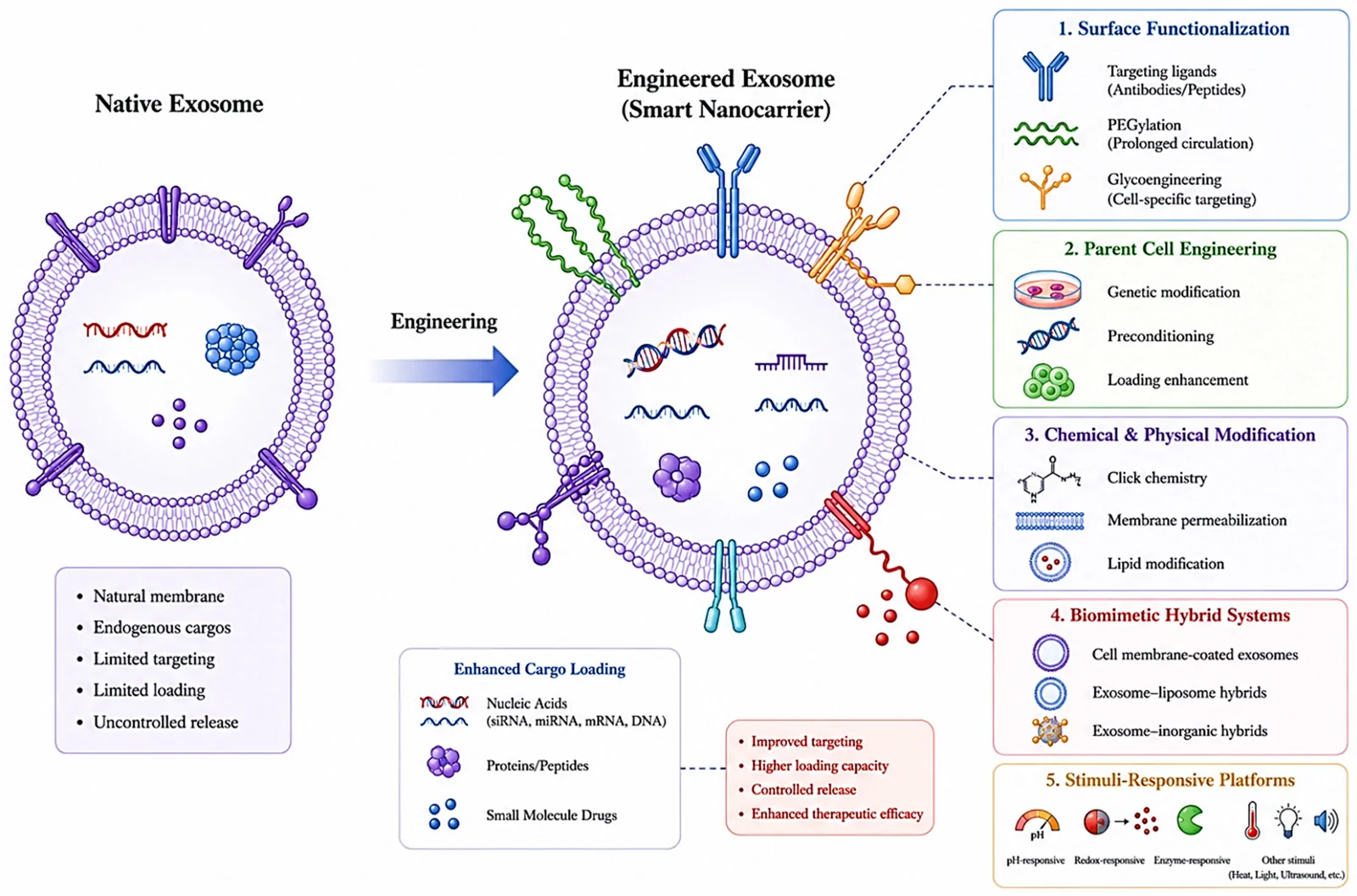
Engineering strategies for the development of smart exosome-based nanocarriers for precision cancer therapy.

Therapeutic applications of native exosomes may be limited by heterogeneous cargo composition, variable targeting efficiency, limited loading capacity for certain therapeutic agents, and challenges associated with reproducible manufacturing [121]. Accordingly, engineered exosomes have been developed through approaches such as cargo loading, surface functionalization, and donor-cell modification to improve targeting, cargo delivery, and therapeutic functionality. In parallel, EV-based systems encompass broader extracellular-vesicle preparations that may be investigated for therapeutic delivery without establishing a specific exosomal subtype. Synthetic exosome-mimetic and hybrid systems, including platforms generated by combining exosomal/EV components with synthetic nanocarriers, represent a distinct class designed to integrate selected biological and physicochemical properties of these systems [122]. Various EV and exosome engineering strategies have been developed to improve targeting, cargo loading, stability, controlled release, and therapeutic functionality. For native exosomes, engineering approaches include surface functionalization, donor-cell modification, and post-isolation cargo loading. Broader EV-based systems may undergo similar engineering procedures when the vesicle population is not defined as exosomes. In contrast, hybrid and exosome-mimetic systems involve the incorporation or fusion of biological vesicle components with synthetic materials and therefore represent distinct platforms rather than modified native exosomes. These approaches have different physicochemical characteristics, manufacturing requirements, and levels of experimental validation, as summarized in Table 5. Native exosomes possess intrinsic biocompatibility and natural intercellular communication capabilities but exhibit limited targeting efficiency and cargo-loading capacity. Through surface functionalization, targeting ligands, antibodies, peptides, aptamers, or polyethylene glycol (PEG) are introduced onto the exosomal membrane to enhance tumor-specific recognition and prolong systemic circulation. Parent cell engineering genetically or physiologically modifies donor cells, enabling the secretion of exosomes enriched with desired therapeutic molecules or membrane proteins. Chemical and physical modification techniques further improve membrane permeability, cargo encapsulation, and structural stability through covalent conjugation, lipid insertion, electroporation, sonication, or extrusion. Biomimetic hybrid systems, generated by fusing exosomes with synthetic liposomes or cell membranes, combine the biological advantages of exosomes with the high loading capacity and functional versatility of artificial nanocarriers. Stimuli-responsive engineering enables controlled drug release in response to tumor-associated stimuli such as acidic pH, elevated glutathione concentrations, overexpressed enzymes, heat, light, or ultrasound (Figure 3). Collectively, these engineering strategies produce advanced exosome-based nanocarriers with enhanced targeting precision, improved therapeutic cargo delivery, and superior antitumor efficacy for next-generation precision cancer therapy.

### 5.1. Native EVs

Exosome surface engineering is one of the most frequently used techniques for enhancing the targeting specificity of exosomes. Exosomes naturally demonstrate certain tropism towards particular target tissues, however, this specific targeting can be improved by adding certain targeting ligands or other molecules, such as peptides, antibodies, aptamers, or small molecules, to the exosome membrane that will interact with receptors overexpressed on the cancer cells’ surface [132]. Moreover, in addition to the active targeting process, surface modification also increases the stability of exosomes, increases their circulation time, and decreases their rapid elimination from the body through the mononuclear phagocyte system. Novel methods of ligand conjugation on exosomes using bioorthogonal chemistry, such as click chemistry and lipid incorporation techniques, provide a mechanism for binding ligands stably onto exosome membranes without damaging them [133].

### 5.2. Donor-Cell/Genetically Engineered EVs

**Figure 4:**
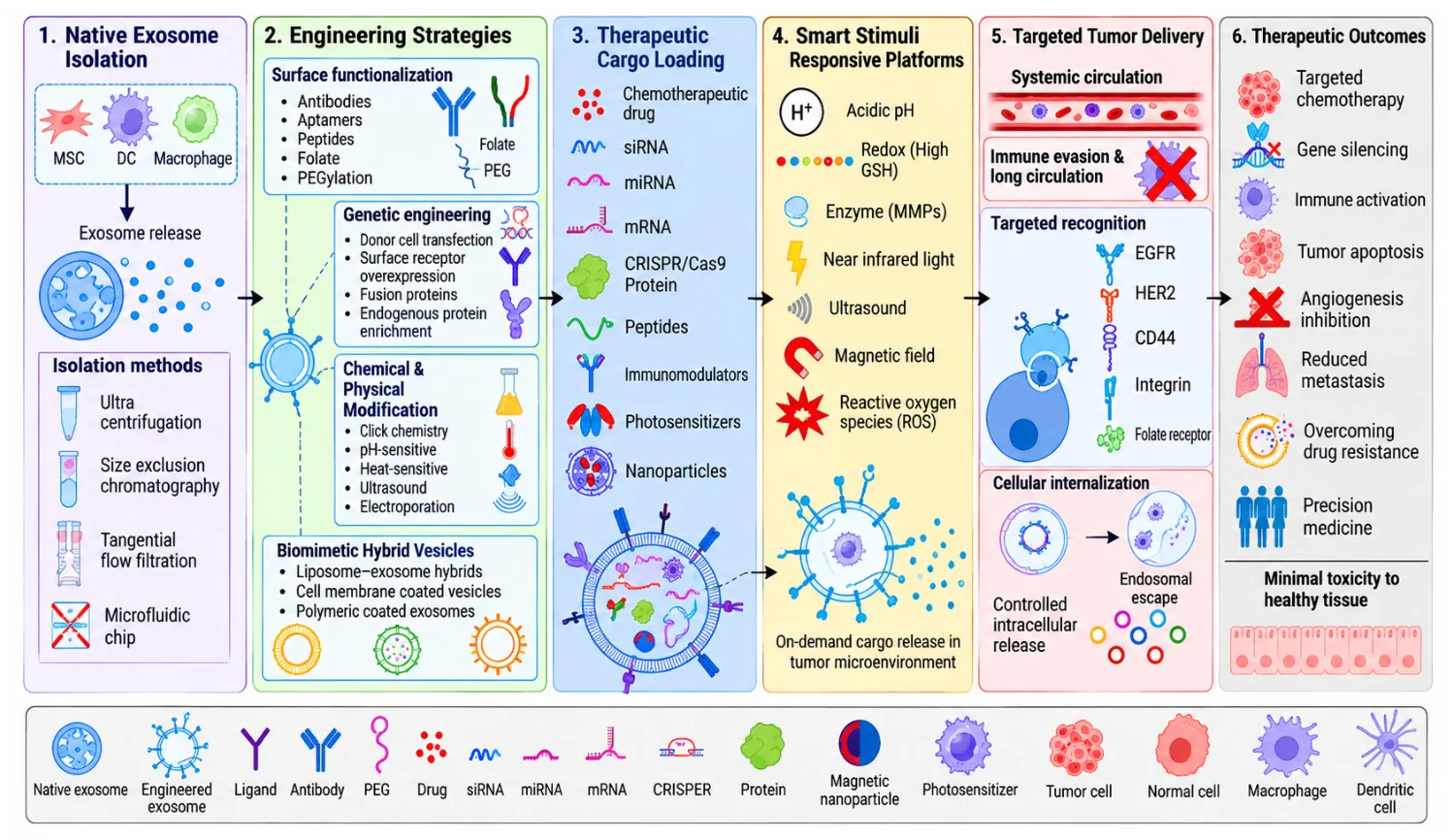
Schematic representation of the engineering methods used to construct smart exosome-based nanocarriers for cancer treatment.

Genetic engineering of donor cells offers a feasible route to fabricate customized therapeutic exosomes. Specifically, parental cell lines are genetically modified by the introduction of plasmids, viruses, or CRISPR-based instruments to specifically enrich exogenous proteins, peptides, or nucleotides into shed exosomes. This in-cell fabrication method retains exosome structures and enables the serial production of functionalized exosomes [134]. Mechanistically, engineered donor cells can express and package targeting or therapeutic proteins that are normally loaded into intraluminal vesicles (ILVs) during exosome formation. By targeting tumor cells with fusion of targeting peptides and membrane proteins, including CD63, CD81, or Lamp2b, receptor-mediated cancer targeting was achieved [135]. Loading with tumor suppressive miRNAs, siRNAs, or immunostimulatory proteins was also observed to promote better anti-tumor activity of the engineered exosomes in many cancer models [136]. Engineered techniques have changed native exosomes to be very effective in delivering drugs owing to surface modification, genetic engineering, cargo loading, and biomimetic techniques. A brief description of such engineered techniques is illustrated in Figure 4.

### 5.3. Post-Isolation Modification

Exosomes after isolation are also amenable to engineering for changes in cargo and composition of exosomes without need for modifying the donor cells. Chemical conjugation involves binding molecules such as targeting moieties, probes for fluorescence imaging, polymers, or therapeutics to the exosomal membrane, either through covalent or non-covalent bonds. For example, PEGylation (e.g., the addition of polyethylene glycol chains to exosomes) enhances colloidal stability and circulation time in vivo [137]. Physical methodologies of therapeutic cargo encapsulation are also frequently adopted and encompass electroporation, sonication, extrusion, freeze-thaw cycles, and membrane permeabilization strategies. The electroporation method allows for a temporary increase in membrane permeability to encapsulated contents, such as nucleic acids. Ultrasound and extrusion allow better loading of hydrophobid compounds. Although many improvements have been reported using these procedures to increase loading, further modifications are needed to protect exosome bioactivity [138,139].

### 5.4. Biomimetic Hybrid Vesicle Systems

Biomimetic hybrid vesicles consist of biological vesicles, such as exosomes, which carry the benefits of natural biological exosomes and the structural benefits of synthetic particles. The vesicles can be produced via the fusion process of exosomes and liposomes/polymeric nanoparticles and possess improved loading capacity, increased half-life, and improved stability [140]. Compared with native vesicles, hybrid exosomes exhibit enhanced advantages, such as increased production yield and enlarged cargo loading capacity. Moreover, through integrated imaging probes, magnetic nanoparticles, and responsive polymers, they are capable of concomitant drug delivery and dynamic therapeutic monitoring. This class of multifunctional platforms demonstrates encouraging outcomes in targeted chemotherapy, gene therapy, and combinational cancer therapy, paving the way for their further clinical translation [141].

### 5.5. Stimuli-Responsive and Smart EV Platforms

Engineering exosomes to be sensitive to stimuli-responsive therapeutic drugs can be specifically unloaded by tumors through their unique biochemical or physiological signals. Owing to the clear distinction of microenvironmental characteristics between tumors and noncancerous tissue, including acidity/alkalinity, oxidation and reduction potential, enzyme levels, and hypoxia, smart exosomes can utilize these and ensure controlled loading/release of the drug and reduce side effects [142].

#### 5.5.1. pH-Responsive Systems

Solid tumors usually display a highly acidic extracellular matrix (pH 6.5–6.8), providing a favorable opportunity for site-specific drug delivery. For example, by functionalizing engineered exosomes with pH-sensitive polymers or acid-cleavable linkers, the therapeutic payload can be protected from degradation in circulation, yet can be efficiently released in the TME (acidic pH). This can increase intracellular drug delivery and the efficiency of cytotoxic chemotherapy and lower the toxicity of the drug [143].

#### 5.5.2. Redox-Responsive Systems

Intracellular levels of glutathione (GSH) are much higher in tumor cells compared to those in healthy cells. Redox-responsive exosomes utilize this concentration disparity by having disulfide bond-containing linkers that are not susceptible to breaking in the extracellular environment but are readily cleaved inside the cell. This allows for the extremely fast intracellular release of the loaded drugs and is especially well suited for the delivery of cancer drugs and gene-editing components [144].

#### 5.5.3. Enzyme-Responsive Systems

Overexpression of enzymes (MMPs-2 and MMP-9, hyaluronidase, cathepsins, and phospholipases), that play important roles in ECM degradation and tumor invasion and growth, is commonly observed in many tumor tissues. In enzyme-responsive exosomes, specific enzymes of tumor tissues cleave enzyme-sensitive peptide linkers and subsequently release encapsulated drugs at the targeted tumor sites, thus providing a higher concentration in the tumor site while minimizing damage to the surrounding normal tissues. This combination is likely to improve the efficacy and safety of future precision cancer therapeutics [145].

## 6. Therapeutic Cargo Loading Approaches

The therapeutic efficacy of exosomes is mainly due to their ability to effectively encapsulate and deliver various bioactive molecules without disrupting vesicle integrity and biological activity. Unlike other nanocarriers, exosomes have a natural phospholipid bilayer that protects the therapeutic cargo from enzyme-induced breakdown, increases its circulation time in the bloodstream, and promotes efficient delivery to intracellular targets. However, native exosomes have a low capacity for cargo encapsulation, requiring the implementation of an optimized loading method aimed at increasing the encapsulation rate without damaging the membrane structure. Two types of approach can be used to load cargo molecules into exosomes: endogenous and exogenous [146,147]. As exosome engineering continues to evolve and improve, the range of therapeutic cargo that can be transported to treat cancer has been significantly widened. Whether it is traditional chemotherapeutic drugs, RNA-based therapy, or gene-editing tools, engineered exosomes serve as an ideal platform for drug delivery. All the various types of therapeutic cargo and their uses are highlighted in Table 6.

### 6.1. Passive and Active Loading Techniques

**Figure 5:**
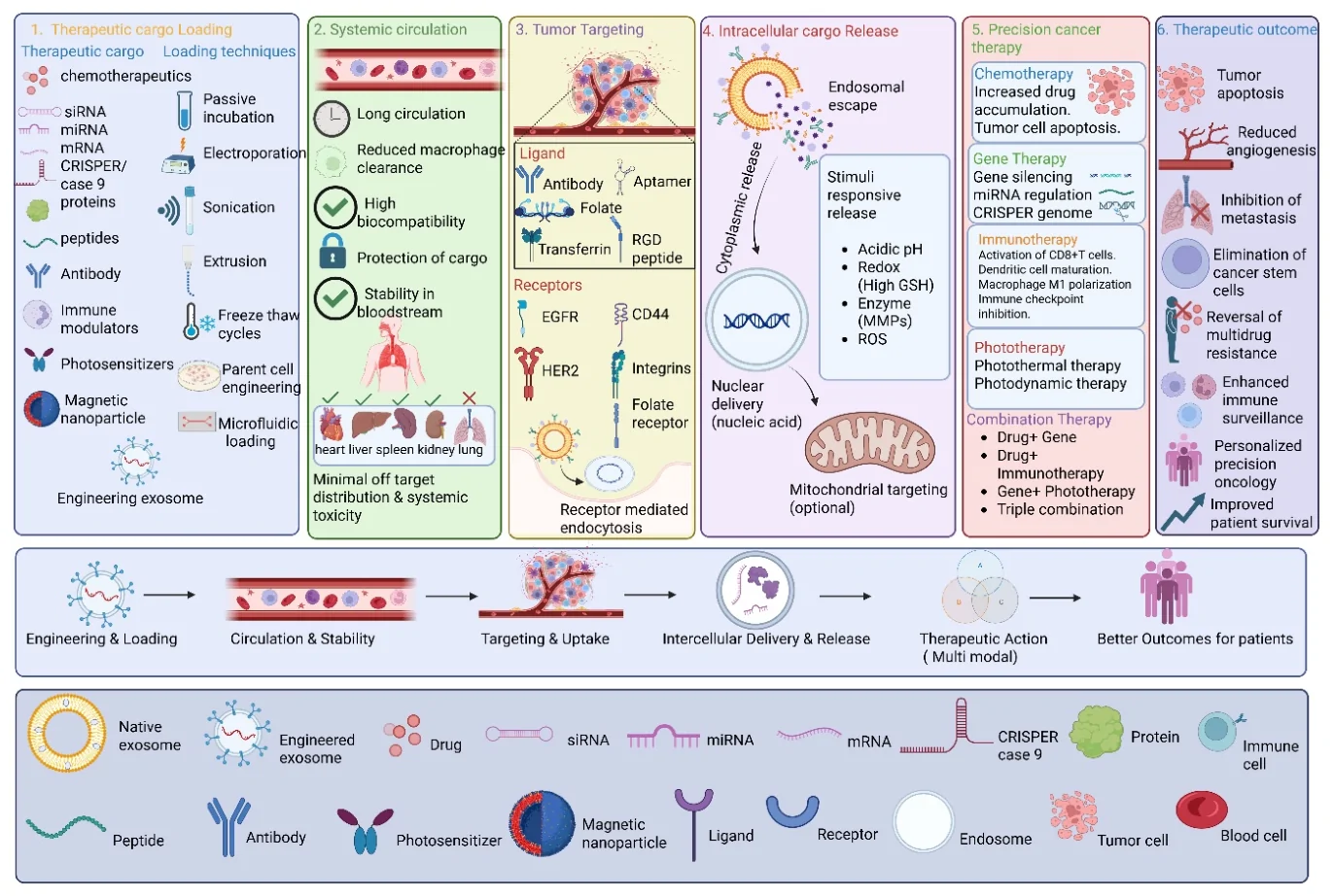
Therapeutic cargo loading and delivery to target tumors by engineered exosomes for precise cancer treatment, which encompasses strategies for cargo loading, transport through the body’s bloodstream, targeted delivery to the tumor, release of cargo into cells, and therapeutic applications using multiple approaches.

There are two types of cargo loading methods: passive and active loading methods. Passive loading uses the simple incubation of therapeutic molecules with purified exosomes or donor cells and depends on the lipid bilayer and concentration gradient. Hydrophobic drugs can be passively loaded by permeating into the lipid bilayer based on the concentration gradient. This technique does not affect the membrane integrity of exosomes and is relatively easy to carry out, however, it has lower encapsulation efficiencies in general, especially for hydrophilic drugs and nucleic acids [163]. Active loading methods, such as electroporation, sonication, extrusion, freeze-thawing, and saponin-based permeabilization, disrupt transient membrane permeability to load the cargoes efficiently into vesicles. For example, electroporation is a popular method to load both siRNA and miRNA, while sonication and extrusion are favorable for loading lipophilic chemotherapeutics. Different EV cargo-loading strategies vary in their suitability for specific cargos, loading efficiency, effects on EV integrity, and overall reproducibility. These approaches range from relatively mild passive loading to physical, membrane-based, and donor-cell engineering strategies, each with distinct advantages and limitations for therapeutic applications, as highlighted in Table 7 below. Although these methods show dramatically higher encapsulation efficiency, excessive disruption of the vesicle membrane can lead to changes in morphology or aggregation of the cargo inside the vesicles. Choosing the proper loading method requires consideration of the encapsulation efficiency and maintenance of vesicle activity [164,165]. Exosome modification has provided an effective way to incorporate multiple therapeutic cargoes to deliver them into tumor tissues with potentially enhanced target recognition and minimal systemic toxicity. The following schematic depiction provides an outline for loading and delivery of these therapeutic cargoes in Figure 5.

### 6.2. Loading of Small-Molecule Chemotherapeutics

Exosomes have shown amazing therapeutic potential for conventional chemotherapy agents. They increase tumor specificity and minimize the adverse effects associated with conventional methods. Various chemotherapy agents, such as doxorubicin, paclitaxel, cisplatin, curcumin, gemcitabine, and methotrexate loaded with engineered exosomes, can be easily prepared by simple passive loading, electroporation, or sonication [174]. The drug-loaded exosomes accumulate in the tumor region after systemic infusion owing to their inherent membrane proteins and longer circulation to circumvent the biological barriers of the artificial nanodelivery system [175]. A few studies using exosome delivery for cancer treatment have indicated that this method can improve the therapeutic index of anticancer drugs through prolonged intracellular accumulation and diminished off-target toxicities. In addition, exosomes have demonstrated their ability to overcome multidrug resistance by bypassing multidrug resistant pumping and directly targeting the drug delivery to the cytoplasm of cancer cells [20].

### 6.3. Nucleic Acid Delivery

**Figure 6:**
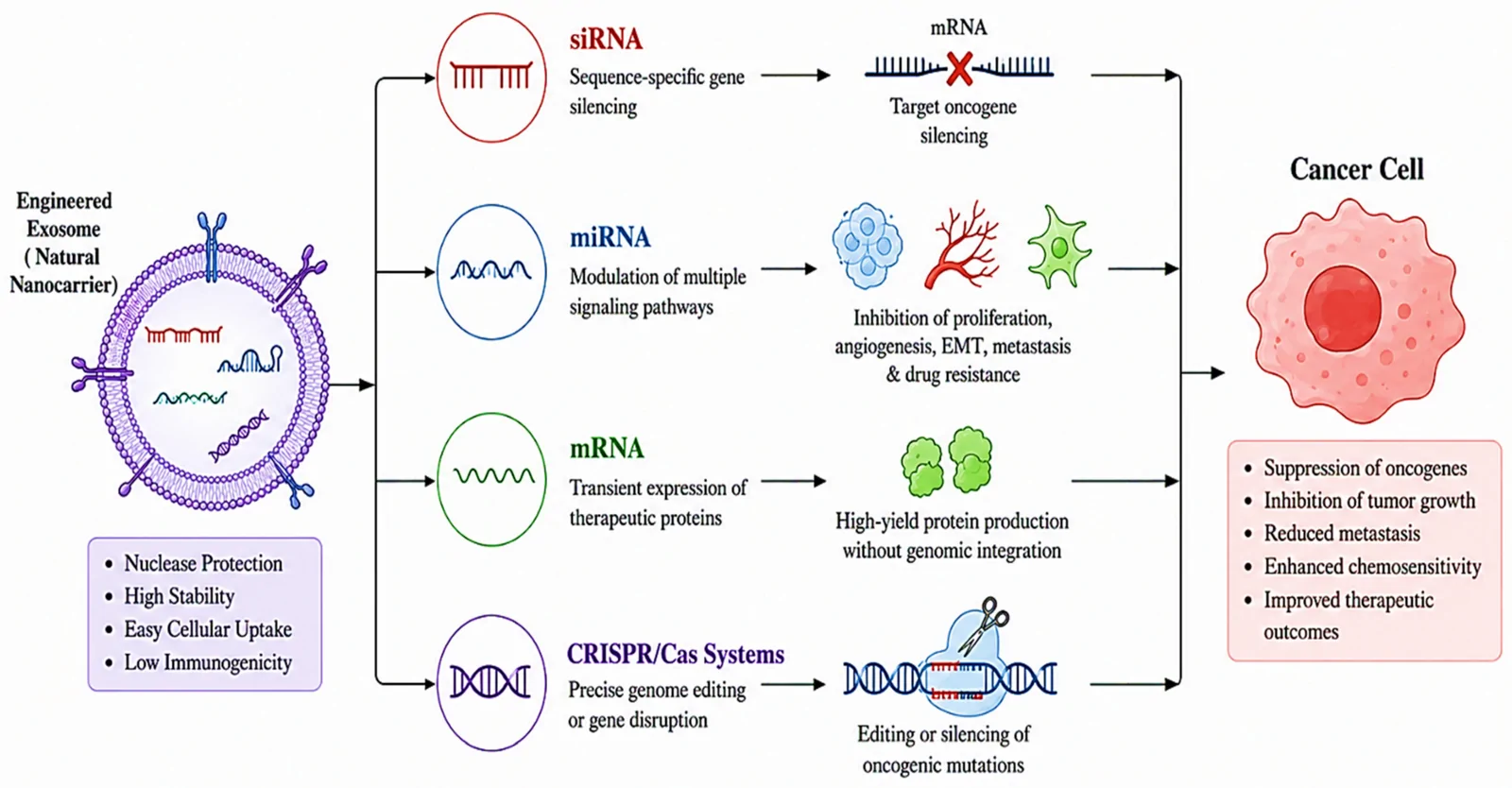
Exosome-mediated nucleic acid cargo delivery for precision cancer therapy.

The natural ability of exosomes to preserve nuclease-resistant, intact nucleic acids makes them potent gene therapeutics’ carriers. The membrane structure allows easy uptake into cells and the delivery of cytoplasmic contents into cells, thus enabling the manipulation of disease pathogenic genes with low immunogenicity [176]. Figure 6 illustrates the role of engineered exosomes as natural nanocarriers for nucleic acid-based cancer therapy. Exosomes encapsulate diverse therapeutic nucleic acid cargos, including siRNA, miRNA, mRNA, and CRISPR/Cas components, protecting them from enzymatic degradation during systemic circulation while facilitating efficient uptake by target cancer cells. Following intracellular delivery, siRNA induces sequence-specific silencing of oncogenes, miRNA regulates multiple signalling pathways involved in proliferation, angiogenesis, epithelial-to-mesenchymal transition (EMT), metastasis, and drug resistance, mRNA enables transient expression of therapeutic proteins without genomic integration, and CRISPR/Cas systems achieve precise genome editing or disruption of oncogenic mutations. Collectively, these mechanisms promote targeted gene regulation, inhibit tumor growth and metastasis, overcome therapeutic resistance, and improve the overall efficacy of precision cancer treatment while minimizing off-target toxicity.

#### 6.3.1. siRNA

Delivery of small interfering RNA (siRNA) via exosomes provides targeted and sequence-specific silencing of genes driving oncogenesis and tumor growth. The use of exosomes for siRNA delivery overcomes the shortcomings associated with the use of synthetic carriers and is associated with enhanced stability and circulation time, as well as greater intracellular targeting, leading to better gene silencing and limited off-target effects [177].

#### 6.3.2. miRNA

The ability of exosomes to carry numerous signalling-modulating miRNAs concurrently could therefore facilitate the delivery of a set of miRNAs that would collectively suppress various oncogenic processes. The loading of exosomes with tumor-suppressive miRNAs, including miR-34a and let-7, has already been exploited for antitumor therapy, resulting in the inhibition of cell proliferation, angiogenesis, epithelial-to-mesenchymal transition, and metastasis, as well as the regaining of sensitivity to chemotherapeutics in drug-resistant tumor [178].

#### 6.3.3. mRNA

Recent interest in mRNA-packaged exosomes has been generated as a convenient delivery vehicle for the temporary production of therapeutic proteins. Exosomes offer numerous advantages over traditional viral methods, such as the absence of genomic integration and prevention of insertional mutagenesis. Exosomes can translate mRNA in the cell to achieve high protein quantity and have been explored for personalized therapies and cancer immunotherapy [179].

#### 6.3.4. CRISPR/Cas Systems

Exosomes in tandem with CRISPR/Cas genome editing tool technology have been at the forefront of achieving high precision oncology. The design of functionalized exosomes may simultaneously deliver both CRISPR Cas protein/guide RNA/CRISPR plasmids into cancer cells, thereby editing or silencing specific oncogenic mutations with very high efficiency. In contrast with viral delivery methods, exosomes-based CRISPR platforms offer superior biocompatibility, lower immunogenicity, and the possibility of multiple dose administration, however, they still need optimization for efficiency and scalability [180,181].

### 6.4. Protein and Peptide Delivery

Beyond nucleotides and small-molecule drugs, engineered exosomes and other EV-based systems have been investigated for the delivery of proteins, peptides, enzymes, cytokines, and antibodies. These therapeutic molecules may be incorporated into or associated with the vesicle structure, where the lipid bilayer can provide protection against enzymatic degradation and support their stability during biological transport. In particular, engineered exosomes displaying apoptotic proteins, immune cytokines, or antitumor-targeting peptides have demonstrated therapeutic potential in several cancer models [182]. Technological improvements in the area of protein engineering have made highly efficient and selective targeting, imaging, and subsequent intracellular drug delivery based on targeting specific tumor molecules displayed on the exosome surface possible. Such multifunctional exosomes are not only capable of delivering drugs in vesicles but also simultaneously perform imaging and immunomodulatory purposes. However, there are challenges to improve the efficiency of protein loading, stability, and developing reliable large-scale production processes required for their clinical application [137,183].

## 7. Exosome-Based Precision Cancer Therapy

The emergence of precision oncology has resulted in a shift from a general approach in cancer therapy to personalized treatment based on the molecular characteristics of every tumor. In this regard, the emergence of precision oncology has resulted in a shift from general therapeutic approaches toward personalized treatment based on the molecular characteristics of individual tumors. In this context, native EVs and exosome-enriched preparations have been investigated as biological nanocarriers for cancer therapy because of their ability to interact with recipient cells and transport diverse therapeutic cargos [184]. Native exosomes do not inherently possess universal or tumor-specific targeting ability, and their tissue distribution and cellular uptake depend on donor-cell characteristics, surface composition, administration route, and biological context [185]. Consequently, engineered exosomes may incorporate targeting peptides, antibodies, aptamers, or other ligands to modify their surface properties and potentially improve tumor-cell recognition and therapeutic delivery, although these effects require experimental validation [186]. Broader EV-based systems may provide similar delivery functions but should not be assumed to have the same biological properties as purified or experimentally characterized exosomes. Synthetic exosome-mimetic and hybrid systems constitute a separate category in which biological vesicle components are combined with synthetic materials to obtain selected delivery or functional characteristics. These platform differences should be considered when interpreting evidence for exosome- and EV-based precision cancer therapy [187,188].

### 7.1. Targeted Chemotherapy

While chemotherapy continues to play an important role in cancer therapy, its efficiency is often limited due to low tumor selectivity, side effects on normal cells, rapid elimination of the drug from the body, and multi-drug resistance. Engineered exosomes represent a biological carrier that can effectively deliver chemotherapeutic drugs to cancer cells without damaging normal tissues. The natural surface proteins of exosomes promote internalization through cell receptor-mediated mechanisms [189]. Doxorubicin, paclitaxel, cisplatin, gemcitabine, and curcumin are some of the anticancer agents that have been effectively loaded into exosomes through passive loading, electroporation, and sonication. Exosome surface modification with targeting ligands, such as folic acid, RGD peptides, transferrin, and HER2 antibodies, facilitates the selective uptake of exosome-encapsulated drugs by cancer cells overexpressing these receptors. Experiments on laboratory animals have revealed that exosome-assisted chemotherapy increases drug efficacy by increasing their bioavailability, decreasing cardiotoxicity and nephrotoxicity, and overcoming the problem of drug resistance to some extent [190].

### 7.2. Gene Therapy and RNA Therapeutics

Gene therapy has become an attractive approach for combating cancer owing to its selective regulation of oncogenic pathways that cause cancer formation and drug resistance. Nevertheless, the application of nucleic acid-based therapy in clinics faces several challenges, such as low stability, degradation, poor cellular uptake, and off-target effects. In contrast, engineered exosomes bypass these obstacles by encapsulating nucleic acids in their lipid membrane and delivering them into cells using physiological endocytosis processes [191]. The use of exosomes has already been proven successful in delivering siRNA, miRNA, mRNA, antisense oligonucleotides, and CRISPR/Cas systems for genetic manipulation. The therapeutic delivery of miRNAs with tumor-suppressive functions, such as miR-34a and let-7, and siRNAs aimed at KRAS, EGFR, BCL-2, and mutant p53 genes has shown successful suppression of oncogene signalling and restoration of sensitivity to chemotherapy [177].

### 7.3. Immunotherapy Applications

The advent of immunotherapy has been an impressive development in cancer therapeutics, whereby the body’s immune system can identify and kill cancer cells. However, its effectiveness is often hampered by immune evasion, lack of sufficient immune cell infiltration, toxicity, and a TME that suppresses immune function. Engineered exosomes have turned out to be effective carriers for immunotherapy because of their ability to communicate between cells and deliver immunomodulatory molecules in a selective way [192]. Exosomes have the capability of stimulating antigen presentation, activation of cytotoxic immune cells, overcoming immune suppression, and enhancing the effectiveness of immunotherapies currently in use depending on their cellular origins and the kind of contents. The great biocompatibility and low immunogenicity, along with their capacity to deliver targeted cargoes, make exosomes different from traditional synthetic nanocarriers [193].

#### 7.3.1. Cancer Vaccines

Thus, exosome-based cancer vaccines can be regarded as a new strategy for cancer treatment, through which it is possible to generate a powerful immune response against tumor cells. Exosomes differ from regular vaccines in their ability to already possess MHC, TAAs, heat shock proteins, and co-stimulatory molecules necessary for antigen presentation to dendritic cells and activation of CD8^+^ cytotoxic T-lymphocytes and CD4^+^ helper T-cells. Dendritic cell-derived exosomes are particularly promising because of the possibility of duplication of the functions of their parent cells with improved stability and easier storage [194,195].

#### 7.3.2. CAR-T Modulation

While CAR-T cell therapy is tremendously effective against certain haematological cancers, its applicability in solid tumors is limited by insufficient infiltration, antigen heterogeneity, antigen escape, and immunosuppressive tumor microenvironment. Herein, exosome engineering has been developed to improve CAR-T cell function and decrease toxicity [196]. CAR-T cell-derived exosomes carry cytotoxic components, such as perforin, granzyme B, and Fas ligand (FasL). This allows exosomes to induce the apoptosis of cancer cells without the need for live CAR-T cells. Exosomes have fewer side effects than whole-cell-based immunotherapy because they are less immunogenic, more able to penetrate tissues, and do not cause cytokine release syndrome or graft-versus-host disease. Furthermore, engineered exosomes can be used to deliver cytokines, stimulatory RNAs, and signalling molecules to enhance CAR-T cell survival and functionality in the tumor microenvironment [197].

#### 7.3.3. Immune Checkpoint Regulation

The PD-1/PD-L1 and CTLA-4 immune checkpoint pathways are critical means by which cancer cells escape immune surveillance. PD-L1 is commonly expressed by exosomes secreted from tumor cells, allowing them to inhibit T-cell activity even when they are at remote locations from the tumor site. Exosome-mediated immune evasion is gaining importance as an immune resistance mechanism [198]. Engineered exosomes represent an appealing approach to address this issue because they deliver anti-PD-L1 siRNA, PD-L1 antibodies, immunostimulatory RNAs, and CRISPR/Cas genetic editing components into tumor tissue sites. The use of such approaches decreases the level of checkpoint molecules and improves T-cell cytotoxicity and cancer immune elimination with minimal adverse effects on the patient’s body. In addition, the use of exosome-mediated checkpoint modulation together with chemotherapeutic agents, radiation therapy, and cancer vaccines has yielded synergistic outcomes that have improved immune antitumor responses [199].

### 7.4. Photothermal and Photodynamic Therapy

Photothermal and photodynamic therapy (PTT and PDT) have evolved as minimally invasive techniques that selectively eliminate tumor cells, leaving the adjacent normal cells unharmed. Nevertheless, there are several factors that limit the effectiveness of PTT/PDT methods, including poor retention of the photosensitizer molecules, inefficient tumor penetration, and non-selective distribution. Engineered exosomes provide an effective solution to all these issues, acting as a natural nanocarrier system for the selective delivery of phototherapeutic molecules [200]. PTT involves exosomes containing components such as gold nanoparticles, graphene oxide, black phosphorus, and polydopamine that can effectively transform NIR light into localized heat and cause irreversible damage to tumor cells. In contrast, PDT involves exosomes containing photosensitizers, such as chlorin e6 (Ce6) and porphyrins, whose activation under laser illumination causes the production of ROS that cause oxidative stress and mitochondrial dysfunction, resulting in apoptosis. Recent findings have demonstrated that a combination of either PTT or PDT along with chemotherapy or immunotherapy can improve the immune response against tumors, inhibit metastasis, and increase treatment efficacy [201,202].

### 7.5. Sonodynamic and Chemo-Dynamic Therapy

Sonodynamic therapy (SDT) and chemo-dynamic therapy (CDT) represent novel therapeutic approaches that take advantage of the physicochemical properties of the tumor microenvironment (TME) for selective treatment of cancer. Unlike traditional phototherapies, SDT relies on low-intensity ultrasound to allow deeper penetration into tissues, thus allowing the treatment of difficult-to-access or deep tumors. Exosome engineering has greatly enhanced the stability, targeting ability, and efficacy of sonosensitizers and catalytic nanomaterials [203,204]. According to SDT, activation of exosome-encapsulated sonosensitizers through exposure to ultrasound induces excess production of reactive oxygen species, causing oxidative stress and subsequent apoptosis of tumor cells. For CDT, the engineered exosomes carry catalytic nanoparticles that induce Fenton or Fenton-like reactions through the use of endogenous hydrogen peroxide in tumors to form very toxic hydroxyl radicals. This process leads to the selective killing of cancer cells without any injury to normal cells. Recent studies indicate that the combination of SDT and CDT with chemotherapy, immunotherapy, and gene therapy leads to synergistic therapeutic effects and helps to combat tumor hypoxia and multidrug resistance [205,206].

### 7.6. Clinical Translation of EV/Exosome-Based Cancer Therapeutics

Several EV- and exosome-based therapeutic approaches have progressed from preclinical research to early clinical investigation in cancer. Clinical studies have explored approaches involving therapeutic cargo loading, immune modulation, and engineered EVs in different cancer types (Table 8). However, most reported studies remain at early clinical stages, and further evidence is required to establish their therapeutic efficacy, safety, biodistribution, and manufacturing consistency. Representative clinical studies are summarized in Table 8.

## 8. Challenges and Future Perspectives

**Figure 7:**
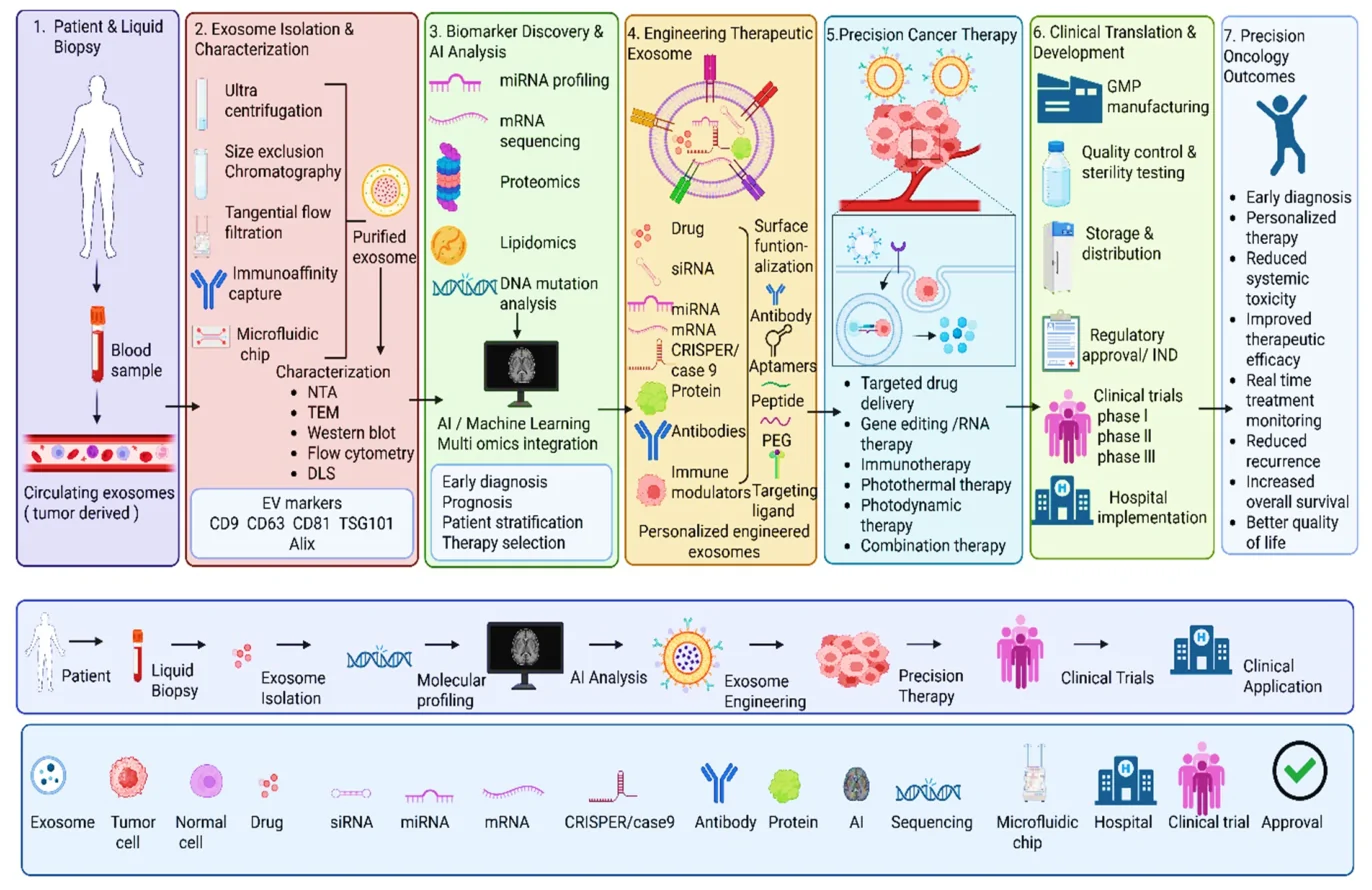
A roadmap showing the clinical translation pipeline for engineered exosomes as precision medicine for oncology, covering procedures from liquid biopsy and exosome isolation to exosomal profiling and biomarker discovery, to engineering therapeutic exosome, clinical-grade precise oncology therapy, clinical investigation and personal application for cancer care.

EV heterogeneity represents a major challenge for the reproducibility and clinical translation of EV-based therapeutics. Variability in donor-cell source, culture conditions, EV isolation, size distribution, molecular cargo, and post-isolation processing can result in differences in EV composition and biological activity. Such variability may affect cellular uptake, biodistribution, therapeutic efficacy, toxicity, and pharmacokinetic behavior. In addition, batch-to-batch differences may complicate dose standardization, quality control, and comparison of results across studies. Therefore, systematic characterization and standardized manufacturing and processing procedures are essential for improving the consistency and translational potential of EV-based therapies. Despite notable advances in exosome modification, there are still a number of issues that impede the clinical implementation of these particles. The first and foremost issue is the absence of standardization of procedures for exosome isolation, purification, characterization, and storage, thus causing inconsistencies in purity, yield, and biological activity. Another challenge related to this area is the impossibility of obtaining GMP-grade exosomes at a large scale because of poor yield, inconsistency between batches, and difficulty in maintaining therapeutic efficacy. Finally, efficient loading and delivery of a cargo pose additional problems, as the currently employed strategies can disrupt membrane integrity. Heterogeneity is also another challenge because the difference in size, content, and sources of these exosomes affects the results of the therapy. Characterization using advanced technologies is thus critical to ensure that they will have the desired effects on patients. Another challenge includes understanding their biodistribution, immune response, pharmacokinetics, and regulation before clinical use. Future research must emphasize the incorporation of artificial intelligence, multi-omics approaches, CRISPR technology, biomimetic hybrid vesicles, and responsive exosomes for enhancing the targeting and effectiveness of treatment. The use of engineered exosomes along with gene therapy, immunotherapy, phototherapy, and nanotechnology can help surmount the issue of tumor heterogeneity and drug resistance. Advances in scale-up processes and their quality control are expected to ensure that engineered exosomes emerge as the future of smart nanoparticles in treating cancer. The process by which engineered exosomes progress from bench-top studies to clinical practice includes various aspects of translational medicine such as liquid biopsy, molecular profiling, drug delivery engineering, clinical study, and regulatory clearance. The flow of translation for this purpose is illustrated in Figure 7.

### 8.1. EV Heterogeneity and Its Impact on Therapeutic Translation

EV heterogeneity represents a major challenge for the reproducibility and clinical translation of EV-based therapeutics. EV populations can differ in their cellular origin, biogenesis, size distribution, surface composition, molecular cargo, and biological activity [207]. Such heterogeneity may arise from differences in donor cell type and physiological state, cancer subtype, culture conditions, hypoxia, therapeutic stimulation, isolation and purification procedures, and post-isolation processing. Consequently, EV preparations obtained under different experimental or manufacturing conditions may exhibit distinct molecular and functional characteristics [208].

Heterogeneity can directly affect therapeutic efficacy because differences in EV surface molecules and cargo composition may influence cellular uptake, tissue distribution, intracellular trafficking, and biological activity. Variations in EV populations may also contribute to differences in pharmacokinetic and biodistribution profiles and may influence the safety or toxicity of a given preparation [209]. From a reproducibility perspective, heterogeneity can contribute to batch-to-batch variation in particle concentration, size distribution, cargo content, surface markers, and functional potency. Differences in isolation and purification methods may further alter the composition of the final EV preparation and complicate comparisons between studies. These issues can make it difficult to establish consistent dose definitions, potency assays, and quality-control specifications [210].

EV heterogeneity also presents challenges for large-scale manufacturing and regulatory translation. Standardized donor-cell conditions, culture parameters, isolation procedures, characterization methods, and storage conditions are required to minimize uncontrolled variability [211]. Comprehensive characterization should ideally include physicochemical properties, molecular composition, purity-related attributes, and functional potency. Establishing reproducible manufacturing and quality-control criteria will therefore be essential for improving consistency and supporting the development of clinically translatable EV-based products [212].

### 8.2. Manufacturing, Storage, and Quality Control of EV-Based Therapeutics

The clinical translation of EV-based therapeutics requires reproducible manufacturing processes that can generate sufficiently large quantities of EVs while maintaining consistent physicochemical and biological characteristics [213]. Large-scale production remains challenging because EV yield and composition are influenced by donor-cell type, cell culture conditions, passage number, culture medium, cell density, and physiological or pathological state [214]. Transitioning from laboratory-scale production to larger manufacturing systems may therefore alter EV yield, composition, and functional properties. Scalable culture platforms, controlled culture conditions, and standardized production parameters are required to minimize such variability [215].

Purification is another critical step because EV preparations may contain proteins, lipoproteins, protein aggregates, cellular debris, and other extracellular particles. Different isolation and purification approaches can produce differences in EV recovery, purity, size distribution, and molecular composition [216]. Therefore, purification methods should be selected according to the intended application while balancing recovery, purity, processing time, scalability, and preservation of EV functionality. Combining complementary purification approaches may improve product quality, although additional processing can also increase manufacturing complexity and potential EV loss [217].

Batch-to-batch variability represents an additional challenge for EV-based products. Variations in donor-cell characteristics, culture conditions, EV isolation, purification, cargo composition, and processing parameters may result in differences in particle concentration, size distribution, surface markers, molecular cargo, and biological activity [9]. Storage and stability are also important considerations because EV characteristics may change during storage depending on temperature, formulation, storage duration, freeze–thaw exposure, and other processing conditions [218].

Quality control should include complementary physicochemical, molecular, and functional assessments. Physicochemical characterization may include particle concentration, size distribution, morphology, and surface-associated markers, whereas molecular characterization can assess relevant RNA, protein, lipid, or therapeutic cargo components [219]. Importantly, characterization of EV identity alone is insufficient to establish therapeutic quality. Functional potency assays should therefore be developed according to the intended mechanism of action and should demonstrate a reproducible biological response relevant to the therapeutic application [220].

## 9. Conclusions

EVs, particularly exosome-enriched and engineered EV-based systems, have emerged as promising platforms for the delivery of therapeutic cargos in cancer. Their biologically derived membranes, complex molecular composition, and ability to interact with recipient cells provide opportunities for targeted delivery of drugs, nucleic acids, proteins, and other therapeutic agents. Advances in surface functionalization, cargo-loading strategies, donor-cell engineering, stimuli-responsive systems, and hybrid approaches have further expanded the potential applications of EV-based nanocarriers in precision cancer therapy.

Despite these advances, several challenges continue to limit the translation of EV-based systems into clinical applications. Biological and methodological heterogeneity, variability in cargo composition, differences in isolation and purification methods, limited loading control, batch-to-batch variability, and difficulties in scalable manufacturing remain important concerns. In addition, comprehensive characterization, quality control, biodistribution, pharmacokinetics, safety evaluation, and regulatory standardization are required to establish consistent therapeutic performance.

Future research should, therefore, focus on developing standardized manufacturing and characterization procedures, improving reproducibility and scalability, optimizing cargo-loading and targeting strategies, and conducting well-designed preclinical and clinical studies. Integrating molecular engineering with advanced characterization and quality-control approaches may help improve the consistency and therapeutic utility of EV-based nanocarriers. Overall, EV-based systems represent a promising but still developing approach for precision cancer therapy, and further rigorous validation is necessary to establish their safety, efficacy, and clinical applicability.

## Figures and Tables

**Table 1:** Classification and biological characteristics of EVs.

EV Subtype	Biogenesis/Origin	Typical Characteristics	Commonly Associated Markers	Molecular Cargo	Primary Biological Functions	Nomenclature/Identification Consideration	Ref.
Exosomes/endosome-derived EVs	Formed within the endosomal system through intraluminal vesicle formation and released following fusion of multivesicular endosomes with the plasma membrane	Generally small EVs with overlapping size distributions; size alone is not sufficient for identification	CD9, CD63, CD81, ALIX, TSG101 and other EV-associated proteins	miRNA, mRNA, lncRNA, circRNA, proteins, lipids, DNA	Cell communication, immune modulation, angiogenesis, drug delivery	The term “exosome” should preferably be used when an endosomal origin is experimentally supported. Common markers and size alone cannot definitively establish exosomal identity	[34]
Small EVs (sEVs)	Biogenesis may not be experimentally established	Small EV populations obtained after isolation or separation	Various EV-associated proteins	miRNA, mRNA, lncRNA, circRNA, proteins, lipids, DNA	Cell communication, immune modulation, therapeutic delivery	Preferred term when small EVs are experimentally isolated but their specific biogenesis cannot be demonstrated	[35]
Microvesicles/ectosomes	Outward budding and fission of the plasma membrane	Heterogeneous size distribution	Integrins, selectins and other variable EV-associated proteins	miRNA, mRNA, proteins, lipids, DNA	Cell communication, coagulation, immune modulation and intercellular signalling	Size and marker profiles alone cannot unambiguously distinguish microvesicles from other EV populations	[36]
Apoptotic bodies	Generated during apoptotic cell disassembly	Generally larger and heterogeneous vesicular structures	Variable; may contain cellular and membrane-associated components	DNA, RNA, proteins, organelles and other cellular components	Clearance of apoptotic material, intercellular communication and immune regulation	Identification should consider apoptotic context rather than relying solely on size or molecular markers	[37]

**Table 2:** Major sources and potential consequences of heterogeneity in extracellular vesicle preparations.

Source of Heterogeneity	Examples/Contributing Factors	Potential Effect on EV Characteristics	Possible Therapeutic Consequences	Ref.
Donor cell type	Mesenchymal cells, tumor cells, immune cells, epithelial cells	Differences in proteins, lipids and nucleic-acid cargo	Altered targeting, uptake and therapeutic activity	[63]
Donor physiological state	Cell activation, proliferation, differentiation, cellular stress	Changes in EV production and cargo composition	Variable biological responses and efficacy	[64]
Cancer subtype	Genetic and phenotypic differences among tumors	Distinct molecular cargo and surface characteristics	Differences in tumor targeting and therapeutic response	[65]
Culture conditions	Medium composition, serum supplementation, cell density, passage number and culture duration	Changes in EV yield, size and cargo	Reduced reproducibility between studies	[66]
Hypoxia	Low-oxygen culture conditions	Altered EV release and molecular cargo	Changes in angiogenesis, tumor interaction and therapeutic activity	[67]
Therapeutic treatment	Exposure to anticancer drugs or other treatments	Treatment-dependent changes in EV composition	Possible changes in efficacy, toxicity and biological activity	[68]
Isolation procedure	Ultracentrifugation, SEC, precipitation, ultrafiltration, affinity-based methods	Differences in recovery, purity and EV subpopulation enrichment	Variable therapeutic performance and reproducibility	[69]
Size distribution	Different EV size populations	Differences in surface composition and cargo distribution	Altered cellular uptake and biodistribution	[70]
Cargo composition	miRNA, mRNA, proteins, lipids and other biomolecules	Variation in molecular content and biological activity	Differences in therapeutic efficacy and potential toxicity	[71]
Post-isolation processing	Storage, freeze–thaw cycles, concentration, buffer conditions	Membrane damage, aggregation or cargo degradation	Reduced stability, altered activity and batch variability	[72]

**Table 3:** Comparative characteristics of extracellular vesicle isolation methods.

Isolation Method	Yield/Recovery	Purity	Reproducibility	Suitable Biological Fluids/Samples	EV Subpopulation Enrichment	Major Limitation	Ref.
Differential ultracentrifugation	Moderate–high	Moderate	Moderate	Plasma, serum, cell culture media and other fluids	Limited	Time-consuming; possible aggregation and variable recovery	[103]
Density-gradient centrifugation	Moderate	High	Moderate	Plasma, serum and cell culture media	Better separation based on density	Low throughput and relatively labor-intensive	[104]
Size-exclusion chromatography (SEC)	Moderate	Moderate–high	High	Plasma, serum and cell culture media	Limited size-based separation	Dilution of EV preparations and limited separation of similarly sized particles	[105]
Polymer-based precipitation	High recovery	Low–moderate	Moderate	Plasma, serum, urine and cell culture media	Poor	Co-isolation of proteins and lipoproteins	[106]
Ultrafiltration	High/rapid concentration	Moderate	Moderate	Plasma, serum and cell culture media	Limited size-based enrichment	Membrane fouling and possible membrane-associated EV damage	[107]
Microfluidics	Variable	High/selective depending on platform	High potential	Small-volume blood-derived samples and other biological fluids	High potential for selective enrichment	Limited processing volume and device complexity	[108]
Immunoaffinity capture	Low–moderate	High	High when standardized	Plasma, serum and other fluids	High; marker-specific populations can be enriched	Depends on target markers; may exclude EV subpopulations lacking the selected antigen	[109]
Acoustic separation	Variable	Moderate–high	Emerging	Blood and other biological fluids	Potential for label-free physical separation	Technology remains under development	[110]
Lab-on-chip systems	Variable	Potentially high	High potential	Particularly suitable for small-volume liquid-biopsy samples	Potentially high depending on integrated capture strategy	Fabrication complexity, limited throughput and need for further standardization	[111]

**Table 4:** Comparative Characteristics of EV-Based and Synthetic Nanocarrier Platforms.

Nanocarrier	Composition/Source	Biocompatibility & Immunogenicity	Targeting/Biological Interaction	Cargo & Loading	Manufacturing/Reproducibility	Major Advantages	Major Limitations	Ref.
Native EVs	Naturally released lipid-bilayer vesicles from donor cells	Generally favorable biological compatibility; depends on source and preparation	Natural cell-interaction properties; targeting is source-dependent and not necessarily tumor-specific	Proteins, RNAs, lipids and small molecules; loading may be limited and heterogeneous	Complex isolation and substantial source- and batch-dependent variability	Biological membrane, endogenous cellular interactions, cargo protection	Heterogeneity, variable composition, limited loading control, purification and scalability challenges	[115]
Engineered EVs	EVs modified through donor-cell engineering or post-isolation modification	May retain biological membrane properties; modification can alter biological behavior	Can be enhanced through ligands, genetic engineering, aptamers and other surface modifications	Broad cargo compatibility; engineering can improve loading or targeting	More complex manufacturing and additional process-control requirements	Improved targeting, cargo delivery and multifunctionality	Process complexity, variable engineering efficiency and potential batch variability	[116]
Liposomes	Synthetic phospholipid vesicles	Generally tunable through lipid composition and surface modification	Surface ligands can be incorporated for active targeting	Suitable for hydrophilic and lipophilic cargos; well-established loading approaches	Relatively controlled composition and scalable manufacturing	Defined composition, versatile cargo loading, established formulation approaches	Possible instability, clearance and need for optimized targeting depending on formulation	[117]
Lipid nanoparticles (LNPs)	Ionizable/cationic lipids, helper lipids, cholesterol and lipid-anchored components	Strongly dependent on lipid composition and formulation	Surface properties can be engineered; biological distribution depends on formulation	Particularly suitable for nucleic acids; high loading efficiency can be achieved	Highly engineerable and amenable to controlled formulation processes	Efficient nucleic-acid delivery and formulation control	Formulation complexity, potential inflammatory responses and tissue-distribution limitations	[118]
Polymeric nanoparticles	Biodegradable or synthetic polymers	Depends on polymer composition, degradation products and surface properties	Can be modified with ligands or surface coatings	Suitable for small molecules, proteins and nucleic acids depending on polymer system	High degree of physicochemical control and formulation flexibility	Tunable size, surface properties and controlled release	Potential toxicity or immune interactions depending on polymer; formulation optimization required	[119]
Other synthetic nanocarriers	Dendrimers, inorganic nanoparticles and related systems	Highly dependent on material composition and surface characteristics	Surface functionalization can provide targeting	Broad range of cargo possibilities depending on platform	Generally high physicochemical control; manufacturing depends on material	Multifunctionality, imaging and therapeutic integration	Material-specific toxicity, complex surface chemistry and regulatory considerations	[120]

**Table 5:** Comparative engineering strategies for EV-based drug delivery.

Engineering Strategy	Principle	Common Techniques	Functional Outcome/Efficiency	Advantages	Limitations/Reproducibility Considerations	Major Cancer Applications	Reference
Surface Functionalization	Modification of the EV surface with targeting ligands	Antibodies, aptamers, RGD peptide, folic acid	Enhanced target-cell recognition and selective uptake	High targeting specificity	Complex synthesis; ligand density and orientation may affect reproducibility	Targeted chemotherapy, imaging	[123]
Genetic Engineering	Modification of donor cells to produce EVs containing desired therapeutic or targeting components	CRISPR/Cas9, plasmid transfection, viral vectors	Sustained incorporation/expression of therapeutic components	Enables controlled therapeutic cargo and surface engineering	Difficult manufacturing; donor-cell variability may affect EV composition	Gene therapy, immunotherapy	[124]
Chemical Modification	Covalent or non-covalent modification of EV membrane components	Click chemistry, PEGylation	Improved stability and/or functionalization	Flexible surface modification	Possible alteration of membrane properties; additional processing steps	Drug delivery	[125]
Physical Modification	Temporary or mechanical alteration of EV membranes for cargo incorporation	Electroporation, sonication, extrusion, freeze–thaw	Increased cargo-loading potential	Useful for loading therapeutic molecules and nucleic acids	Possible membrane damage, aggregation, or changes in EV properties	RNA delivery, chemotherapy	[126]
Hybrid/Biomimetic Systems	Fusion of EVs with synthetic nanocarriers or other biomimetic components	Liposome–EV fusion	Increased cargo capacity and multifunctionality	Combines biological and synthetic carrier properties	Manufacturing complexity and potential batch variability	Multifunctional nanomedicine	[127]
Stimuli-Responsive Engineering	Incorporation of systems responsive to tumor-associated stimuli	pH-, redox-, enzyme-responsive systems	Controlled or site-specific cargo release	Potential for tumor-selective release	Early-stage development; requires optimization of response characteristics	Precision oncology	[128]
Lipid Insertion	Incorporation of lipid-conjugated ligands into the EV membrane	DSPE-PEG, cholesterol conjugation	Surface functionalization while potentially preserving membrane structure	Relatively simple modification approach	Moderate ligand stability and possible variability in incorporation	Targeted drug delivery	[129]
Aptamer Surface Functionalization	Decoration of EV surfaces with nucleic-acid aptamers	AS1411, EpCAM aptamers	Enhanced target recognition with potentially low immunogenicity	High targeting specificity	Limited in vivo stability may require further optimization	Breast, colorectal and lung cancer	[130]
Nanoparticle Hybridization	Fusion or incorporation of EVs with inorganic nanoparticles	Gold nanoparticles and related systems	Addition of imaging, targeting or therapeutic functionality	Multifunctional delivery potential	Additional manufacturing and characterization requirements	Combined imaging and therapy	[131]

**Table 6:** Therapeutic cargo loading strategies and their applications in engineered exosome-based cancer therapy.

Therapeutic Cargo	Representative Molecules	Loading Method	Therapeutic Mechanism	Advantages	Major Cancer Applications	Clinical Status	Reference
Chemotherapeutic Drugs	Doxorubicin, Paclitaxel, Cisplatin, Gemcitabine	Incubation, Sonication, Electroporation	Cytotoxicity	Reduced systemic toxicity	Breast, Lung, Ovarian, Pancreatic cancer	Preclinical	[148,149]
siRNA	KRAS, EGFR, BCL-2 siRNA	Electroporation	Gene silencing	Potentially enhanced target recognition	Solid tumors	Preclinical	[150]
miRNA	miR-34a, let-7, miR-122	Electroporation/Transfection	Tumor suppressor restoration	Multi-target regulation	Multiple cancers	Preclinical	[151]
mRNA	Therapeutic mRNA	Endogenous loading	Protein expression	No genomic integration	Cancer immunotherapy	Emerging	[152]
CRISPR/Cas System	Cas9 + sgRNA	Electroporation	Genome editing	Precise gene correction	Precision oncology	Experimental	[153]
Proteins/Peptides	TRAIL, Cytokines, Antibodies	Donor-cell engineering	Immune activation	High biological activity	Immunotherapy	Preclinical	[154]
Long Non-coding RNA (lncRNA)	MEG3, GAS5	Electroporation, Transfection	Regulation of oncogenic gene expression	Long-lasting gene regulation	Breast, Gastric cancer	Preclinical	[155]
DNA Therapeutics	Plasmid DNA	Electroporation	Gene replacement	Stable gene expression	Solid tumors	Experimental	[156]
Small-Molecule Inhibitors	Sorafenib, Erlotinib, Gefitinib	Incubation	Kinase inhibition	Improved tumor targeting	Liver, Lung cancer	Preclinical	[157,158]
Immune Checkpoint Inhibitors	Anti-PD-L1, Anti-CTLA-4	Surface engineering	Immune activation	Reduced systemic toxicity	Melanoma, NSCLC	Preclinical	[159]
Photosensitizers	Chlorin e6 (Ce6), Indocyanine Green (ICG)	Sonication	ROS generation	Enhanced phototherapy	Breast, Glioblastoma	Preclinical	[160]
Sonosensitizers	Titanium dioxide, IR780	Electroporation	Ultrasound-induced ROS production	Deep tissue penetration	Pancreatic, Liver cancer	Experimental	[161]
Enzymes	Catalase, Glucose oxidase	Donor-cell engineering	Tumor metabolic modulation	Enhanced combination therapy	Solid tumors	Experimental	[162]

**Table 7:** Comparative characteristics of EV cargo-loading strategies.

Loading Strategy	Suitable Cargo	Loading Efficiency	Effect on EV Integrity	Main Advantages	Major Limitations	Ref.
Passive incubation	Mainly hydrophobic small molecules	Generally low–moderate	Relatively low disruption	Simple and easy to perform	Limited loading of hydrophilic drugs and nucleic acids	[166]
Electroporation	siRNA, miRNA, mRNA and other nucleic acids	Moderate–high	Possible membrane disruption or aggregation	Useful for nucleic-acid loading	Requires optimization and may affect EV integrity	[167]
Sonication	Small molecules and some macromolecules	Moderate–high	Temporary membrane disruption	Enhanced loading efficiency	Possible structural alterations	[168]
Extrusion	Small molecules and nucleic acids	Moderate–high	Can affect membrane structure	Efficient mixing and loading	Mechanical stress and size alteration	[169]
Freeze–thaw cycles	Proteins, nucleic acids and small molecules	Variable	Potential membrane disruption	Simple and relatively accessible	Repeated processing may affect EV stability	[170]
Membrane permeabilization	Various therapeutic cargos	Variable–high	Depends on permeabilizing agent	Can improve cargo entry	Possible changes in membrane properties	[171]
Endogenous/donor-cell loading	RNA, proteins and other biologically produced cargos	Variable	Generally preserves biologically produced vesicles	Enables incorporation during EV biogenesis	Requires donor-cell manipulation and process optimization	[172]
Genetic engineering	Proteins, RNAs and targeting molecules	Variable	Depends on donor-cell modification	Enables controlled expression and surface engineering	Manufacturing complexity and biological variability	[173]

**Table 8:** Representative clinical studies of EV/exosome-based therapeutic approaches in cancer.

Clinical Trial ID	EV/Exosome Source	Therapeutic Strategy/Cargo	Cancer Type	Phase	Status/Clinical Evidence
NCT01159288	Dendritic cell-derived exosomes	Tumor-antigen-loaded exosomes (Dex2)	Non-small-cell lung cancer	Phase II	Completed; clinical study evaluated safety, feasibility, immune responses and progression-related outcomes
NCT03608631	Mesenchymal stromal cell-derived exosomes	KRAS^G12D siRNA (iExosomes)	Metastatic pancreatic cancer with KRAS^G12D mutation	Phase I	Early-phase clinical evaluation of safety, tolerability, dosing and therapeutic potential
NCT01294072	Plant-derived exosomes	Curcumin-loaded plant exosomes	Colon cancer	Phase I	Clinical study evaluating the delivery and localization of curcumin-containing plant-derived exosomes
NCT05375604	Engineered cell-derived EVs/exosomes	CDK-004 containing a lipid-tagged STAT6 antisense oligonucleotide	Advanced hepatocellular carcinoma and liver metastases from gastric/colorectal cancer	Phase I	Terminated; early clinical development was discontinued before demonstrating clinical efficacy
NCT04592484	Engineered exosomes	exoSTING, a STING agonist-loaded exosome platform	Advanced solid tumors	Phase I/II	Early clinical development; evaluated as an engineered exosome-based immunotherapeutic approach

## Data Availability

No new data were created or analyzed in this study. Data sharing is not applicable to this article.
